# MAGL protects against renal fibrosis through inhibiting tubular cell lipotoxicity

**DOI:** 10.7150/thno.92848

**Published:** 2024-02-04

**Authors:** Shan Zhou, Xian Ling, Jielin Zhu, Ye Liang, Qijian Feng, Chao Xie, Jiemei Li, Qiyan Chen, Shuangqin Chen, Jinhua Miao, Mengyao Zhang, Zhiru Li, Weiwei Shen, Xiaolong Li, Qinyu Wu, Xiaoxu Wang, Ruiyuan Liu, Cheng Wang, Fan Fan Hou, Yaozhong Kong, Youhua Liu, Lili Zhou

**Affiliations:** 1 State Key Laboratory of Organ Failure Research, National Clinical Research Center of Kidney Disease, Guangdong Provincial Clinical Research Center for Kidney Disease, Guangdong Provincial Key Laboratory of Nephrology, Division of Nephrology, Nanfang Hospital, Southern Medical University, Guangzhou, China.; 2 Department of Health Care, Zhongshan Hospital of Xiamen University, School of Medicine, Xiamen University, Xiamen, China.; 3 Nephrology Department, The First People's Hospital of Foshan, Foshan, China.; 4 Division of Nephrology, Department of medicine, The Fifth Affiliated Hospital Sun Yat-Sen University, Zhuhai, China.; 5 School of Pharmaceutical Sciences and School of Biomedical Engineering, Southern Medical University, Guangzhou, China.

**Keywords:** MAGL, lipotoxicity, renal fibrosis, 2-AG, FAO

## Abstract

**Rationale:** Renal fibrosis, with no therapeutic approaches, is a common pathological feature in various chronic kidney diseases (CKD). Tubular cell injury plays a pivotal role in renal fibrosis. Commonly, injured tubular cells exhibit significant lipid accumulation. However, the underlying mechanisms remain poorly understood.

**Methods:** 2-arachidonoylglycerol (2-AG) levels in CKD patients and CKD model specimens were measured using mass spectrometry. 2-AG-loaded nanoparticles were infused into unilateral ureteral obstruction (UUO) mice. Lipid accumulation and renal fibrosis were tested. Furthermore, monoacylglycerol lipase (MAGL), the hydrolyzing enzyme of 2-AG, was assessed in CKD patients and models. Tubular cell-specific MAGL knock-in mice were generated. Moreover, MAGL recombination protein was also administered to unilateral ischemia reperfusion injury (UIRI) mice. Besides, a series of methods including RNA sequencing, metabolomics, primary cell culture, lipid staining, etc. were used.

**Results:** 2-AG was increased in the serum or kidneys from CKD patients and models. Supplement of 2-AG further induced lipid accumulation and fibrogenesis through cannabinoid receptor type 2 (CB2)/β-catenin signaling. β-catenin knockout blocked 2-AG/CB2-induced fatty acid β-oxidation (FAO) deficiency and lipid accumulation. Remarkably, MAGL significantly decreased in CKD, aligning with lipid accumulation and fibrosis. Specific transgene of MAGL in tubular cells significantly preserved FAO, inhibited lipid-mediated toxicity in tubular cells, and finally retarded fibrogenesis. Additionally, supplementation of MAGL in UIRI mice also preserved FAO function, inhibited lipid accumulation, and protected against renal fibrosis.

**Conclusion:** MAGL is a potential diagnostic marker for kidney function decline, and also serves as a new therapeutic target for renal fibrosis through ameliorating lipotoxicity.

## Introduction

The prevalence of CKD is increasing worldwide, with rates ranging from 8% to 16% 1. Tubulointerstitial fibrosis, a common pathological feature of CKD, is a significant risk factor for the high morbidity and mortality associated with the disease 2,3. Unfortunately, early detection of renal fibrosis remains challenging, and effective treatment options are currently lacking 4. Therefore, it is crucial to understand the mechanisms underlying renal fibrosis in order to develop early diagnostic methods and effective therapeutic interventions for CKD. Renal epithelial cell is a major component of kidney. Renal fibrosis is closely associated with tubular cell injury 5-7. These cells have a high metabolic rate and require a significant energy supply. To meet this demand, renal epithelial cells rely on FAO, an oxygen-dependent metabolic process that occurs within the mitochondria, to generate adenosine triphosphate (ATP) 8,9. FAO involves the degradation of fatty acids to produce energy 10,11.

Kidney is the second-highest organ with mitochondrial abundance, which almost exclusively relies on FAO for ATP production 12,13. Lipid droplets play key roles in maintaining lipid balance, but excessive lipid accumulation in cells leads to lipotoxicity, meaning that the accumulation of lipid intermediates or metabolites impairs cell functions 14-16. In renal tubular cells, decreased lipid metabolism results in lipid droplet accumulation and further induces lipotoxicity 17. Maintaining lipid homeostasis in kidney is crucial for meeting energy demands 18. However, in kidney injury, fatty acid utilization is disrupted, leading to lipid accumulation and subsequent lipotoxicity damages, characterized by mitochondrial impairment, endoplasmic reticulum stress, and increased reactive oxygen species production, etc. 19-22. Numerous studies have confirmed that lipotoxicity plays a key role in renal fibrosis 23-25. Key regulators of FAO-related gene transcription are peroxisome proliferator-activated receptor alpha (PPARα) and peroxisome proliferator-activated receptor gamma coactivator-1 alpha (PGC-1α) 23,26,27. Previous researches have demonstrated that decreased levels of PPARα and PGC-1α contribute to FAO dysfunction and the formation of lipid droplets in renal tubular cells, leading to the development of renal fibrosis 23,26,28. Interestingly, studies, including our own, have shown that β-catenin regulates PGC-1α 29. However, the potential role of β-catenin in modulating FAO dysfunction requires further investigation.

The endocannabinoid system (ECS) is composed of receptors, specifically cannabinoid receptor 1 and 2 (CB1 and CB2), endogenous ligands referred to as endocannabinoids (primarily 2-AG and Anandamide), and enzymes responsible for metabolizing these ligands 30. Previous studies have indicated that CB2/β-catenin plays a crucial role in tubular cell injury and renal fibrosis 31,32. Additionally, the downregulation of PGC-1α in aging kidneys has been linked to the ECS 33. These findings suggest a potential involvement of the ECS and its downstream pathways in the dysregulation of FAO. However, further investigation is required to fully comprehend the precise mechanisms. Of note, a previous report showed the level of 2-AG was increased in kidneys of UUO 34, suggesting 2-AG/CB2 could be involved in energy metabolism in renal fibrosis. However, this should be clarified. Furthermore, MAGL is a major enzyme of ECS and responsible for the degradation of 85% of 2-AG 35. MAGL is a 33 kDa serine hydrolase, which can decompose monoacylglycerols into fatty acid and glycerol 36. As a comprehensive metabolic center, MAGL can also regulate lipid signaling transduction 37. However, the role of MAGL has not been elucidated in kidney area.

In this study, we discovered that 2-AG promotes lipid accumulation and fibrogenesis in renal tubular cell through β-catenin signaling. Our findings provide an insightful view of the ECS in the kidney area and suggest MAGL is a promising strategy for treating CKD. We also found MAGL hydrolyzes 2-AG and decreases in clinical patients and CKD models. Supplementation of MAGL effectively ameliorates FAO dysfunction and retards renal fibrosis. MAGL could serve as a diagnostic risk factor for the decline of renal function. Furthermore, MAGL could also serve as an innovative therapeutic strategy for CKD.

## Results

### 2-AG increases in CKD and drives lipid deposition and fibrogenesis in tubular cells

**Figure 1:**
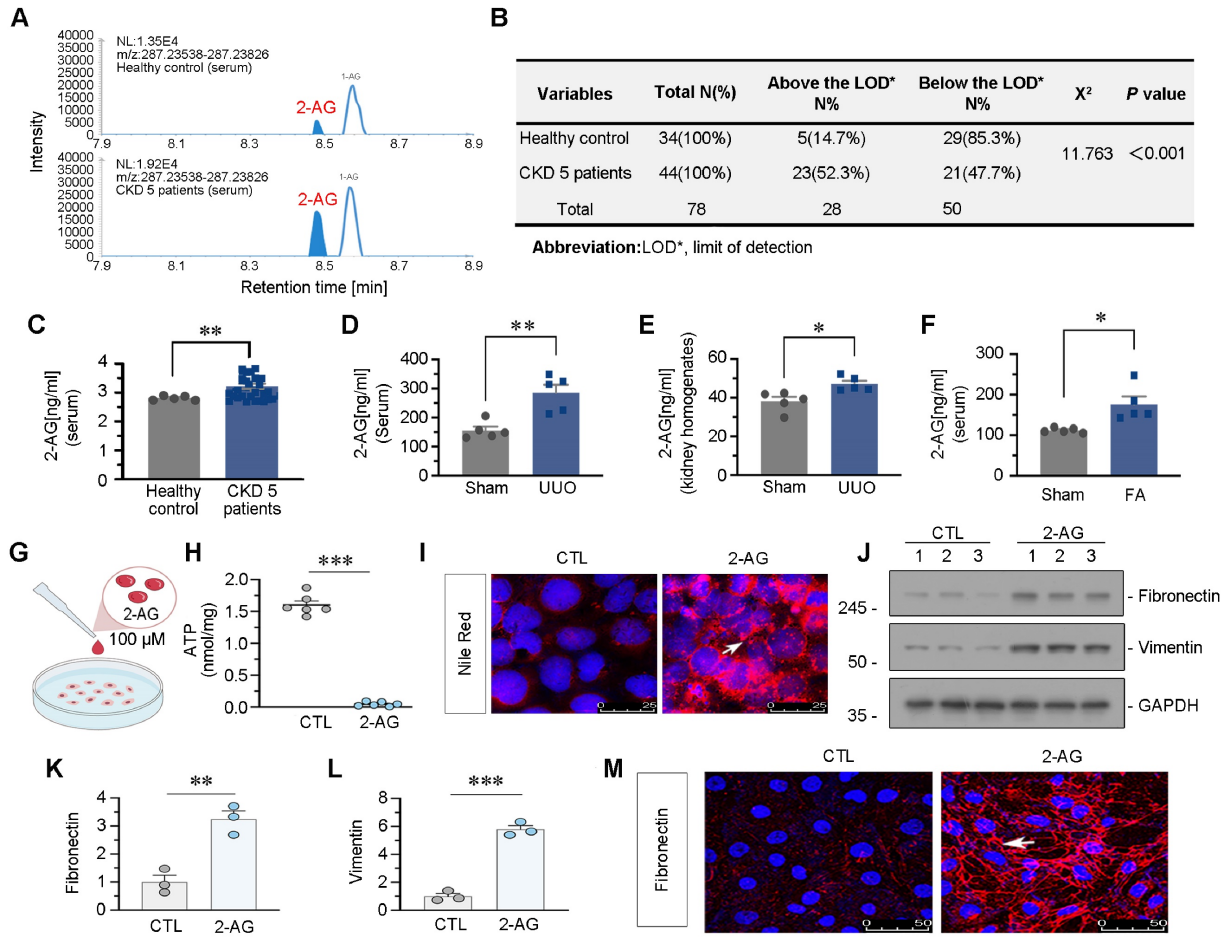
** 2-AG increases in CKD and drives lipid deposition and fibrogenesis in tubular cells. A.** Representative graphs showing 2-AG levels in serum from healthy people and CKD patients at stage 5. 2-AG was identified by liquid chromatograph mass spectrometry (LC/MS) analysis. **B.** Table showing the detectable rate of 2-AG in serum from healthy people and CKD patients at stage 5. **C.** Representative graph showing 2-AG levels in serum from healthy people and CKD patients at stage 5 by LC/MS analysis. **P < 0.01 versus the healthy control group. n = 34 (healthy control); n = 44 (CKD patients at 5 stage). **D-E.** Representative graphs showing 2-AG levels in serum and kidney homogenates by LC/MS analysis. UUO mice were sacrificed at 7 d after surgery. *P < 0.05, **P < 0.01 versus the sham control group alone. n = 5.** F.** Representative graph showing 2-AG levels in serum of FA and sham group of mice, which were identified by LC/MS analysis. *P < 0.05 versus the sham control group alone. n = 5. FA: folic acid.** G.** HK-2 cells were treated with 2-AG (100 μM) for 24 h. **H.** Representative graph showing ATP levels in 2 groups. ***P < 0.001 versus the control group. n = 6. **I.** Representative immunofluorescence micrographs showing the expression of lipid (Nile Red staining) in 2 groups. White arrow indicates positive staining. Scale bar, 25 μm. **J-L.** Representative western blot and quantitative data showing the expression of Fibronectin and Vimentin in 2 groups. Numbers (1-3) indicate each individual culture in a given group. **P < 0.01, ***P < 0.001 versus the control group alone. n = 3. **M.** Representative immunofluorescence micrographs showing the expression of Fibronectin in 2 groups. White arrow indicates positive staining. Scale bar, 50 μm.

We firstly examined the levels of 2-AG in the circulation of 34 healthy individuals and 44 patients with CKD at stage 5. As depicted in Figure 1A-C, serum 2-AG exhibited a significant increase in CKD patients. The chi-square test results further confirmed a higher detection rate of 2-AG in CKD group compared to control group. We also measured 2-AG levels in the serum or kidney homogenates of UUO mice and folic acid nephropathy (FAN) mice, both of which were utilized as CKD models. Significantly elevated levels of 2-AG were observed in both groups compared to control (Figure 1D-F). Subsequently, HK-2 cells, a human proximal tubular cell line, were cultured and subjected to 2-AG treatment (Figure 1G). The findings demonstrated that the administration of 2-AG led to a reduction in ATP production, as illustrated in Figure 1H. Moreover, lipid accumulation was observed upon exposure to 2-AG (Figure 1I). Furthermore, 2-AG elicited fibrogenesis (Figure 1J-M). These findings suggest 2-AG may play a significant role in CKD, influencing disease progression and modulating lipid metabolism, fibrosis, and energy metabolism in renal tubular cells.

### 2-AG promotes lipid deposition and renal fibrosis in UUO mice

**Figure 2:**
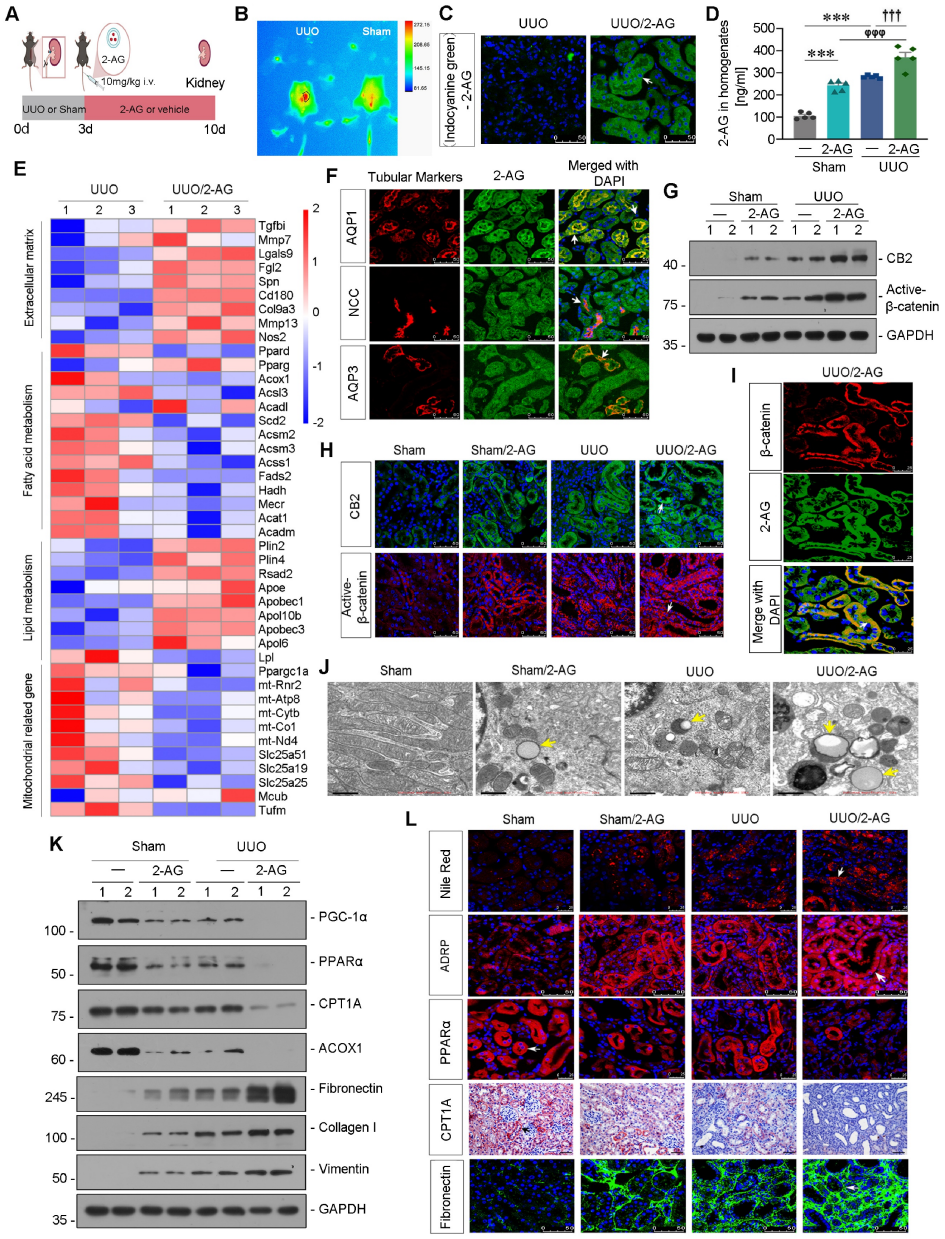
** 2-AG accelerates lipid deposition and renal fibrosis in UUO mice. A.** Experimental design. Mice were performed UUO or sham surgery. Red bar indicates intravenous injections of 2-AG (10 mg/kg/d) or vehicle for 7 d. Mice were sacrificed at 10 d after surgery. **B.** *In vivo* fluorescence image showing 2-AG was enriched in UUO-affected kidney. 2-AG (10 mg/kg) was first labeled with ICG and was injected into UUO or sham mice through tail vein for 2 h. Images were taken from UUO or sham animals by a Bruker Small Animal Optical Imaging System. **C.** Representative micrographs showing the localization of ICG-labeled 2-AG in UUO mice. White arrow indicates positive staining. Scale bar, 50 μm. **D.** Representative graph showing 2-AG levels in kidney homogenates by LC/MS analysis. ^***^P < 0.001 versus the sham control group alone; ^φφφ^*P* < 0.001 versus the 2-AG group alone; ^†††^*P* < 0.001 versus the UUO group alone. n = 5. **E.** Representative heatmap plot of transcriptomic analysis showing the changes in Extracellular matrix, Fatty acid metabolism, Lipid metabolism and Mitochondria-related genes in 2 groups. Numbers (1 - 3) indicate each individual animal in a given group. **F.** Representative micrographs showing colocalization of 2-AG and segment-specific tubular markers in UUO/2-AG mice. Kidney sections were stained for various segment-specific tubular markers (red) by immunofluorescence. The following segment-specific tubular markers were used: proximal tubule, Aquaporin-1 (AQP1); distal tubule, sodium-chloride cotransporter (NCC); and collecting duct, Aquaporin-3 (AQP3). 2-AG was detected by the fluorescence of ICG. White arrows indicate positive tubules with colocalization of 2-AG and specific tubular markers. Scale bar, 50 μm. **G.** Representative western blot showing renal CB2 and Active-β-catenin expressions in different groups. Numbers (1 - 2) indicate each individual animal in a given group. n = 5. **H.** Representative micrographs showing the expression of CB2 (top) and Active-β-catenin (bottom) in 4 groups. White arrows indicate positive staining. Scale bar, 50 μm. **I.** Representative micrographs showing colocalization of β-catenin (red) and 2-AG (green) in UUO/2-AG mice. White arrow indicates positive tubules with colocalization of 2-AG and β-catenin. Scale bar, 25 μm. **J.** Representative transmission electron microscopy (TEM) images showing the lipid droplets in renal tubular epithelial cells in 4 groups. Yellow arrows indicate lipid droplets. Scale bar, 1 μm. **K.** Representative western blot showing renal expression of PGC-1α, PPARα, CPT1A, ACOX1, Fibronectin, Collagen I and Vimentin in different groups. Numbers (1 - 2) indicate each individual animal in a given group. n = 5. **L.** Representative micrographs showing the expression of lipid droplets (Nile Red staining), ADRP, PPARα, CPT1A and Fibronectin in 4 groups. Arrows indicate positive staining. For Nile Red and PPARα staining, scale bar, 25 μm; for ADRP, CPT1A and Fibronectin staining, scale bar, 50 μm.

Liposome-nanoparticle-encapsulated 2-AG was labeled with indocyanine green (ICG) to enhance its aqueous solubility and administered to UUO mice (Figure 2A). In vivo imaging demonstrated a substantial accumulation of 2-AG in UUO-affected kidney 2 h after intravenous injection, whereas sham mice did not exhibit such accumulation (Figure 2B). Fluorescence analysis revealed that 2-AG was predominantly localized in tubules (Figure 2C), rather than in glomeruli (Figure S1A). Additionally, the 2-AG content in kidney homogenates was assessed, revealing a further increase induced by 2-AG in UUO mice (Figure 2D). We then performed RNA sequencing (Figure S1B-C). The heatmap showed 2-AG treatment increased extracellular matrix and lipid accumulation, but decreased fatty acid metabolism and mitochondrial function (Figure 2E). Gene Set Enrichment Analysis (GSEA) also revealed low enrichments of unsaturated fatty acid metabolism and upregulation of lipid complex and extracellular matrix in UUO mice with 2-AG treatment, as shown in Figure S1D-F. The co-staining showed 2-AG could be evolved by all segment of tubules (Figure 2F). We assessed the protein levels of CB2 and Active-β-catenin, and both western blot and immunofluorescence analyses revealed that 2-AG significantly upregulated their levels (Figure 2G-H and Figure S1G). Interestingly, 2-AG was highly colocalized with β-catenin expression (Figure 2I). We further conducted non-targeted metabolomics analysis in kidney tissues in both UUO and UUO/2-AG groups. The results revealed that lipids and lipid-like molecules accounted for the majority of detected substances (Figure S1H). Heatmap analysis demonstrated 2-AG induced a significant increase in medium, long as well as very long-chain fatty acids and their derivatives, such as Palmitic acid, Azelaic acid, Octanedioic acid (Figure S1I). Moreover, the transmission electron microscopy (TEM) analysis showed 2-AG strongly induced lipid accumulation in UUO mice and also induced mild deposition of lipid in sham mice (Figure 2J). We then examined the FAO and fibrosis-related proteins. As shown in Figure 2K and Figure S1J-L, PGC-1α, PPARα, carnitine palmitoyl-transferase 1A (CPT1A), and acyl-CoA oxidase 1 (ACOX1) exhibited downregulation in UUO mice, and their expression was further reduced by 2-AG. Conversely, treatment with 2-AG led to an increased induction of Fibronectin, Collagen I, and Vimentin. Furthermore, fibrogenesis, lipid accumulation, and FAO function decline were also observed in UUO mice and were exacerbated by treatment with 2-AG, as shown by staining of Nile Red, adipose differentiation-related protein (ADRP, the most well-characterized membrane protein associated with lipid droplets 38), PPARα, CPT1A, Fibronectin and Sirius Red (Figure 2L, Figure S1M-N).

### MAGL is decreased in CKD and correlated with preservation of kidney function

**Figure 3:**
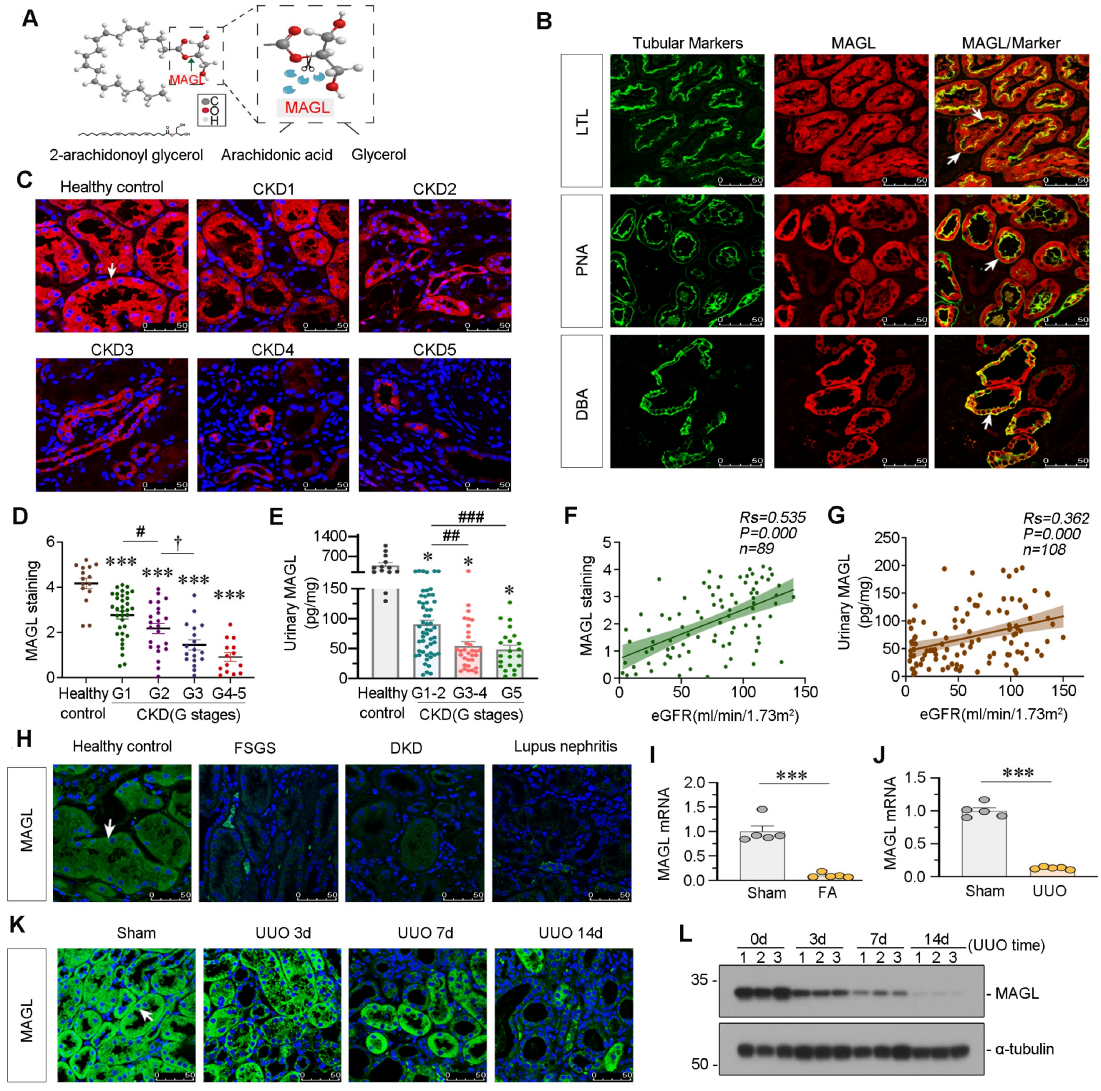
** MAGL is decreased in CKD and correlated with preservation of kidney function. A.** Schematic diagram showing MAGL hydrolyzing 2-AG. The ester bond at the sn-2 position of 2-AG was broken by MAGL to form arachidonic acid (AA) and glycerol. **B.** Representative micrographs showing colocalization of MAGL and various segment-specific tubular markers in kidneys from healthy control. Kidney sections were stained for MAGL (red) and various segment-specific tubular markers (green) by immunofluorescence. The following segment-specific tubular markers were used: proximal tubule, lotus tetragonolobus lectin (LTL); distal tubule, peanut agglutinin (PNA); and collecting duct, dolichos biflorus agglutinin (DBA). White arrows indicate positive tubules with colocalization of MAGL and specific tubular markers. Scale bar, 50 μm. **C.** Representative immunofluorescence micrographs showing the expression of MAGL in healthy control and CKD patients at different stages. White arrow indicates positive staining. Scale bar, 50 μm. **D.** Quantitative data showing quantification of MAGL positive staining in healthy control and CKD patients at different stages. n = 15 (healthy control); n = 34 (patients at CKD stage 1); n = 23 (patients at CKD stage 2); n = 18 (patients at CKD stage 3); n = 14 (patients at CKD stage 4-5). ***P < 0.001 versus healthy control group alone; ^#^*P* < 0.05 versus CKD stage 1 patient group; ^†^*P* < 0.05 versus CKD stage 2 patients group**. E.** Quantitative data showing urinary MAGL in healthy control and CKD patients at different stages. n = 13 (healthy control); n = 54 (patients at CKD stage 1-2); n = 32 (patients at CKD stage 3-4); n = 22 (patients at CKD stage 5). *P < 0.05 versus healthy control group; ^##^*P* < 0.01, ^###^*P* < 0.001 versus CKD stage 1 - 2 patient group. **F.** Linear regression showing the Spearman correlation coefficient (*Rs*) and *P* value between positive staining levels of renal MAGL and eGFR from CKD patients respectively. n = 89. **G.** Linear regression showing the Spearman correlation coefficient (*Rs*) and *P* value between urinary MAGL and eGFR from CKD patients respectively. n = 108. **H.** Representative micrographs showing renal expression of MAGL in healthy control and CKD patients with different etiologies, such as Focal Segmental Glomerulosclerosis (FSGS), Diabetic Kidney Disease (DKD) and Lupus Nephritis. Arrow indicates positive staining. Scale bar, 50 μm. **I.** Quantitative data showing the mRNA level of MAGL in mice with or without intraperitoneal injection of folic acid at 250 mg/kg. Mice were sacrificed at 14 d after injection. ***P < 0.001 versus the sham control group alone. n = 5. **J.** Quantitative data showing the mRNA level of MAGL in UUO and sham mice. UUO mice were sacrificed at 7 d after surgery. ***P < 0.001 versus the sham control group alone. n = 5. **K.** Representative immunofluorescence micrographs showing renal MAGL expression in different groups. Mice were sacrificed at different days after UUO surgery. White arrow indicates positive staining. Scale bar, 50 μm. **L.** Representative western blot showing the expression of MAGL in different groups. Numbers (1 - 3) indicate each individual animal in a given group. n = 5.

MAGL, an enzyme responsible for the hydrolysis of 2-AG (Figure 3A), exhibited a broad expression pattern in renal tubules as evidenced by co-staining with various tubule segment markers such as lotus tetragonolobus lectin (LTL), peanut agglutinin (PNA) and dolichos biflorus agglutinin (DBA) (Figure 3B). In order to further explore its role in kidney, we examined MAGL expression in patients at various stages of CKD. Notably, there was a significant decrease in MAGL levels in tubules as CKD progressed, as evidenced in Figure 3C-D. Additionally, we observed a similar decline pattern in urinary MAGL levels, as shown in Figure 3E. Correlation analysis demonstrated a positive relationship between MAGL expression and preservation of estimated glomerular filtration rate (eGFR) (Figure 3F), as well as a positive correlation between urinary MAGL levels and eGFR (Figure 3G). MAGL was found to be reduced in various types of CKD (Figure 3H). We further investigated the expression of MAGL in animal models. The mRNA levels of MAGL in the kidneys of FAN and UUO mice exhibited a significant decrease (Figure 3I-J). Furthermore, in both UUO mice and adriamycin (ADR) mice models, the protein levels of MAGL exhibited a progressive decline over time, as demonstrated in Figure 3K-L and Figure S2A-D. The results suggest that MAGL serves as a prognostic factor for renal function decline in CKD.

### Loss of MAGL correlates with lipid accumulation and fibrosis

**Figure 4:**
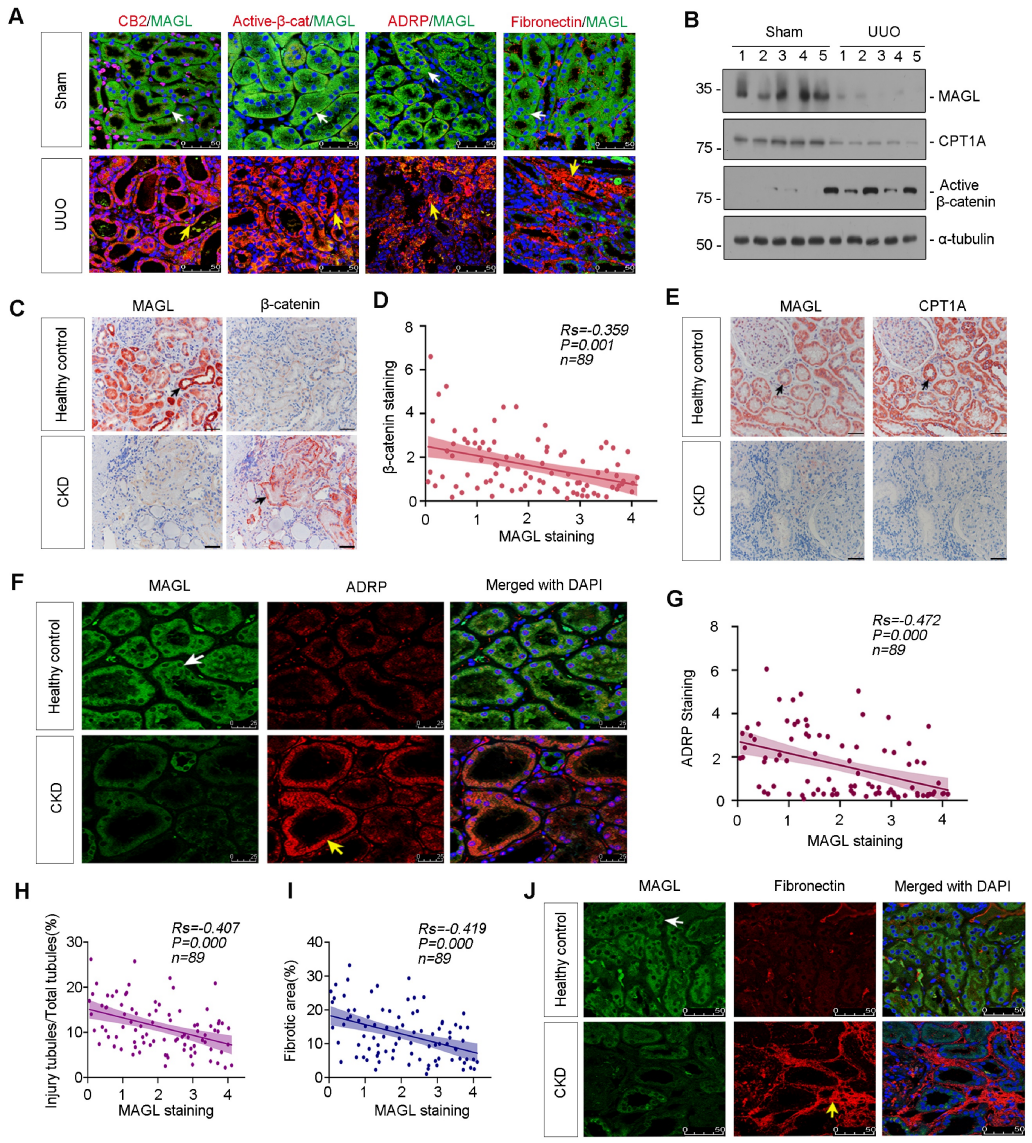
** Loss of MAGL correlates with lipid accumulation and fibrosis. A.** Representative micrographs showing co-staining of MAGL and CB2, MAGL and Active-β-catenin, MAGL and ADRP, and MAGL and Fibronectin in kidneys from sham and UUO mice. Kidney sections were stained for CB2, Active-β-catenin, ADRP, Fibronectin (red) and MAGL (green) by immunofluorescence. Arrows indicate positive staining. Scale bar, 50 μm. **B.** Representative western blot showing the expression of MAGL, CPT1A and Active-β-catenin in UUO and sham mice. Numbers (1 - 5) indicate each individual animal in a given group. n = 5. **C.** Representative micrographs showing renal MAGL (left) and β-catenin (right) expression in healthy control and CKD patient. Black arrows indicate positive staining. Scale bar, 50 μm. **D.** Linear regression showing the Spearman correlation coefficient (*Rs*) and *P* value between positive staining level of MAGL and β-catenin from CKD patients respectively. n = 89. **E.** Representative micrographs showing renal MAGL (left) and CPT1A (right) expression in sequential sections from healthy control and CKD patient. Black arrows indicate positive staining. Scale bar, 50 μm. **F.** Representative micrographs showing co-staining of MAGL and ADRP in kidneys from healthy control and CKD patient. Kidney sections were stained for MAGL (green) and ADRP (red) by immunofluorescence. Arrows indicate positive staining. Scale bar, 25 μm. **G.** Linear regression showing the Spearman correlation coefficient (*Rs*) and *P* value between positive staining levels of MAGL and ADRP from CKD patients respectively. n = 89.** H-I.** Linear regression showing the Spearman correlation coefficient (*Rs*) and *P* value between positive staining levels of MAGL and injury tubules or fibrotic area from CKD patients respectively. n = 89. **J.** Representative micrographs showing co-staining of MAGL and Fibronectin in kidneys from healthy control and CKD patient. Kidney sections were stained for MAGL (green) and Fibronectin (red) by immunofluorescence. Arrows indicate positive staining. Scale bar, 50 μm.

The co-staining revealed a significant decrease in MAGL expression in UUO mice, accompanied by upregulation of CB2, β-catenin activation, ADRP, and Fibronectin (Figure 4A). Western blot analysis further confirmed the substantial reduction of MAGL and CPT1A expression, as well as the induction of Active β-catenin in UUO mice (Figure 4B and Figure S2E-G). Additionally, MAGL and CPT1A were demonstrated their strong expression and co-localization in control kidney, but absent in UUO (Figure S2H). The similar results were also observed in ADR and UIRI mice models (Figure S2I-Q). The correlation between MAGL and β-catenin was examined. When MAGL decreased in CKD, β-catenin increased, and a negative correlation was observed between them (Figure 4C-D). In healthy controls, MAGL widely expressed and co-localized with CPT1A, while in CKD, MAGL and CPT1A decreased (Figure 4E). A negative correlation was also observed between MAGL and ADRP (Figure 4F-G). A negative correlation was found between MAGL and injury tubules or renal fibrosis (Figure 4H-I). Moreover, co-staining of MAGL and Fibronectin showed MAGL-negative tubules were surrounded by Fibronectin-positive area (Figure 4J).

In vitro, MAGL was added to cell culture treated with 2-AG. The results indicated that MAGL reduced 2-AG levels by at least 50% and suppressed CB2 expression while leaving CB1 unaffected (Figure S3).

### MAGL effectively inhibits TGF-β1-induced lipotoxicity and fibrosis in tubular cells

**Figure 5:**
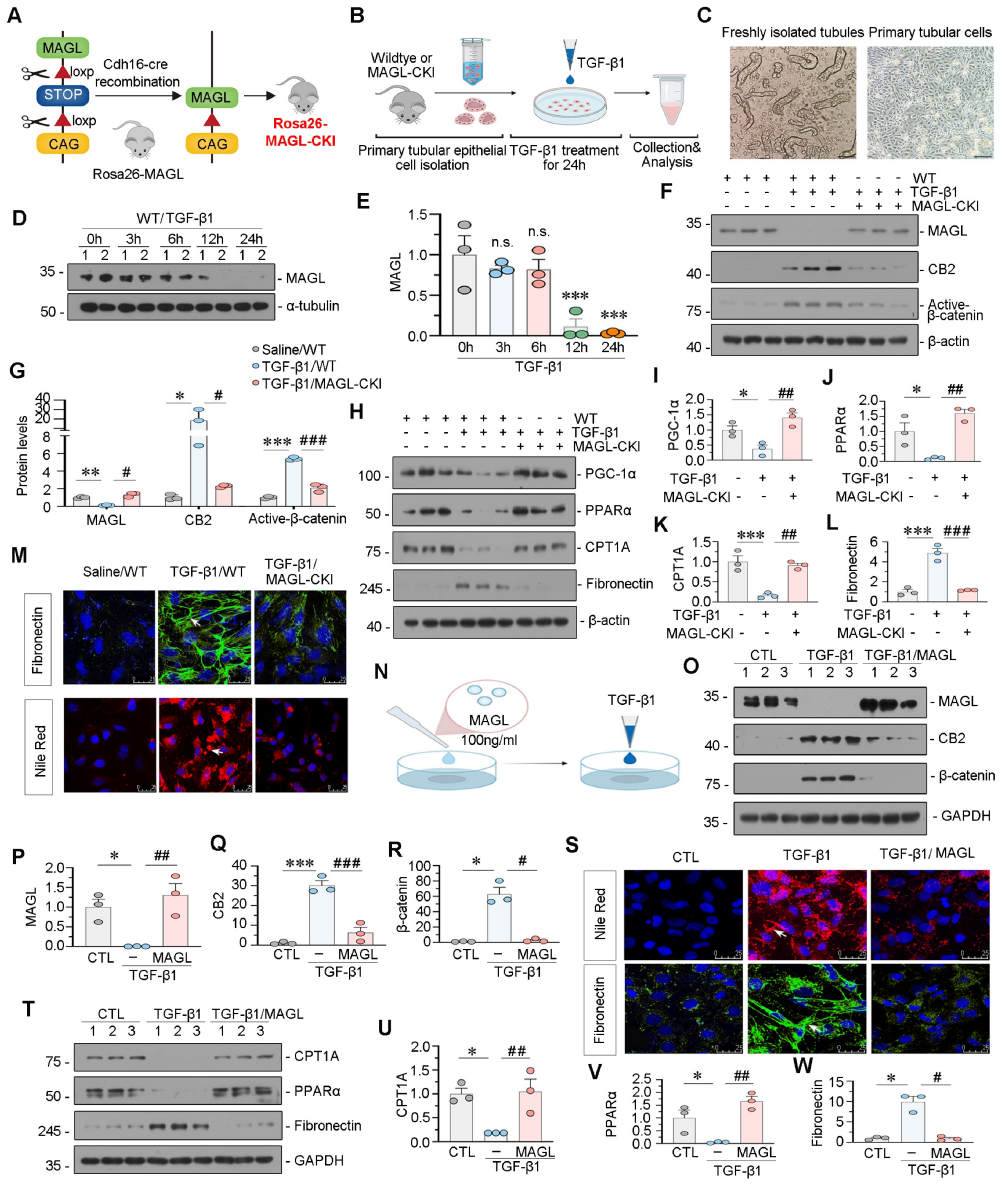
**MAGL effectively inhibits TGF-β1-induced lipotoxicity and fibrosis in tubular cells. A.** Representative graph showing the construction of tubular cell specific MAGL knock-in mice (Rosa26-MAGL-CKI).** B.** Experimental design. Primary tubular epithelial cells were isolated from wildtype mice or MAGL-CKI mice, and then treated with Saline or TGF-β1 (5 ng/ml) for 24 h. **C.** Representative micrographs showing freshly isolated tubules and cultured primary tubular cells in bright field. Scale bar, 100 μm. **D-E.** Representative western blot and quantitative data showing the expression of MAGL in different groups. Primary tubular epithelial cells isolated from wildtype mice were treated with TGF-β1 (5 ng/ml) for indicated time period (0, 3, 6, 12, 24 h). Numbers (1 - 2) indicate each individual culture in a given group. n.s., ***P < 0.001 versus the control group. n = 3. n.s.: none of significance. **F-G.** Representative western blot and quantitative data showing the expression of MAGL, CB2 and Active-β-catenin in different groups. *P < 0.05, **P < 0.01, ***P < 0.001 versus the control group alone; ^#^*P* < 0.05, ^###^*P* < 0.001 versus the TGF-β1 treatment group alone. n = 3. **H-L.** Representative western blot and quantitative data showing the expression of PGC-1α, PPARα, CPT1A and Fibronectin in different groups. *P < 0.05, ***P < 0.001 versus the control group alone; ^##^*P* < 0.01, ^###^*P* < 0.001 versus the TGF-β1 treatment group alone. n = 3. **M.** Representative immunofluorescence micrographs showing the expression of Fibronectin and lipid (Nile Red) in different groups. White arrows indicate positive staining. Scale bar, 25 μm. **N.** HK‐2 cells were pretreated with recombinant MAGL protein (100 ng/ml) for 1 h, and then treated with TGF-β1 (5 ng/ml) for 24 h. **O-R.** Representative western blot and quantitative data showing the expression of MAGL, CB2 and β-catenin in different groups. Numbers (1 - 3) indicate each individual culture in a given group. *P < 0.05, ***P < 0.001 versus the control group alone; ^#^*P* < 0.05, ^##^*P* < 0.01, ^###^*P* < 0.001 versus the TGF-β1 treatment group alone. n = 3.** S.** Representative immunofluorescence micrographs showing the expression of lipid (Nile Red) and Fibronectin in different groups. White arrows indicate positive staining. Scale bar, 25 μm. **T-W.** Representative western blot and quantitative data showing the expression of CPT1A, PPARα, and Fibronectin in different groups. Numbers (1 - 3) indicate each individual culture in a given group. *P < 0.05 versus the control group alone; ^#^*P* < 0.05, ^##^*P* < 0.01 versus the TGF-β1 treatment group alone. n = 3.

We generated tubular cell-specific MAGL knock-in mice (MAGL-CKI) and isolated primary tubular cells from both wildtype and MAGL-CKI mice. Subsequently, these cells were stimulated with TGF-β1 (Figure 5A-C). Our findings revealed a time-dependent reduction in MAGL protein levels in primary tubules of wildtype mice in response to TGF-β1 (Figure 5D-E). Western blot analysis revealed that MAGL-CKI successfully inhibited the increased expression of CB2 and Active-β-catenin induced by TGF-β1 (Figure 5F-G).

Additionally, MAGL-CKI reversed the decrease in FAO levels induced by TGF-β1 and mitigated the expression of Fibronectin (Figure 5H-L). The efficacy of MAGL-CKI in reducing lipid deposition and fibrosis was further validated through fluorescence staining (Figure 5M). In addition to isolate primary cells, we further validated our findings by applying MAGL recombinant protein to HK-2 cells. Significantly, the consistent application of MAGL effectively mitigated TGF-β1-induced alterations, including lipid deposition, fibrosis, activation of CB2/β-catenin, and reduced FAO levels (Figure 5N-W). Similarly, the MAGL inhibitor JZL-184 demonstrated opposite effects (Figure S4).

### Tubular cell specific MAGL knock-in mice inhibit CB2/β-catenin signaling

**Figure 6:**
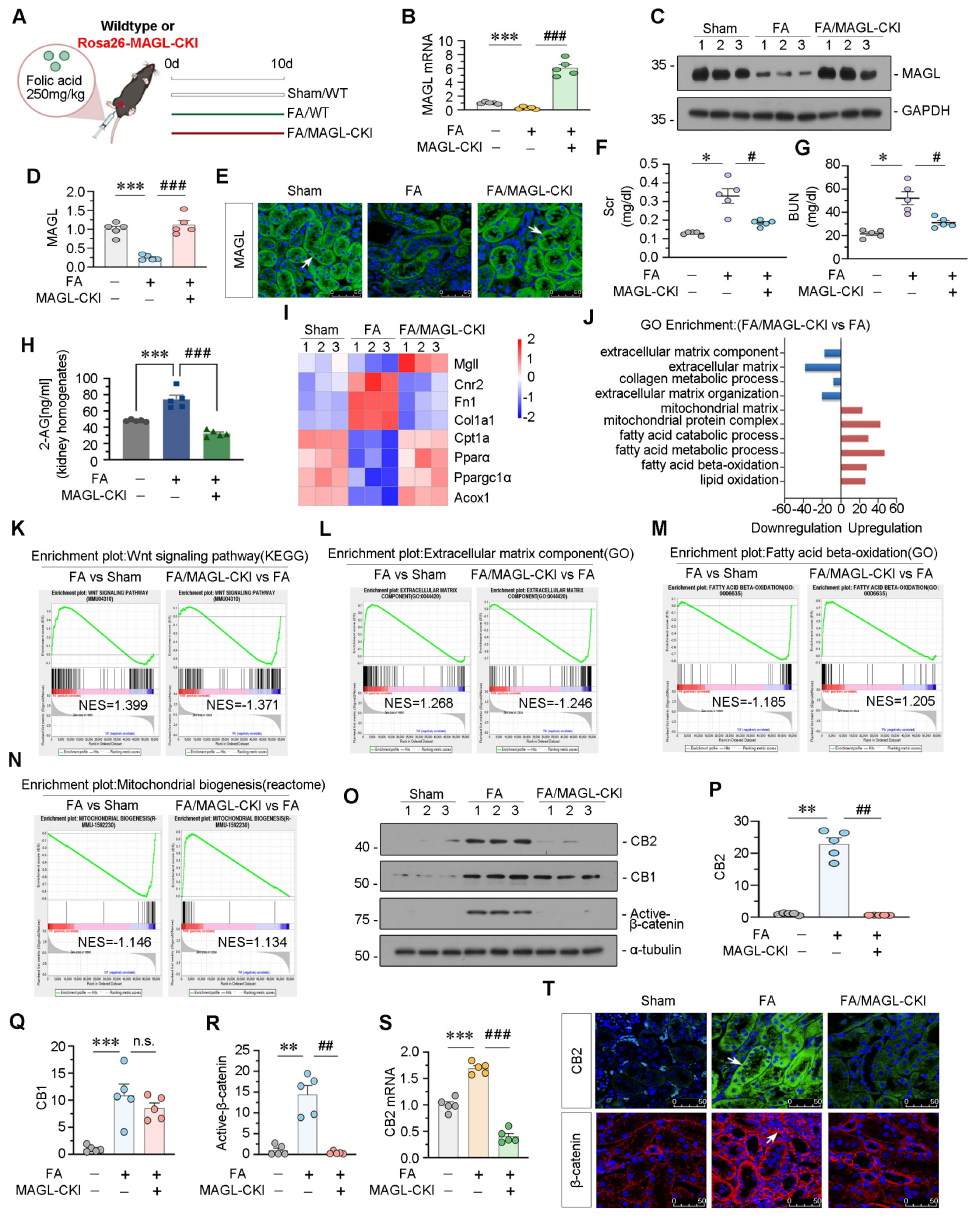
** Specially knock-in of MAGL in tubular cell inhibits CB2/β-catenin signaling in FAN mice. A.** Experimental design. White bar and green bar indicate wildtype mice were administered intraperitoneal injections of saline or folic acid at 250 mg/kg. Red bar indicates Rosa26-MAGL-CKI mice were administered intraperitoneal injections of folic acid at 250 mg/kg. Mice were sacrificed at 10 d after injection. FA: folic acid. MAGL-CKI: tubular cell specific MAGL knock-in mice. **B.** Quantitative data showing renal mRNA levels of MAGL in different groups. ***P < 0.001 versus the wildtype control group alone; ^###^*P* < 0.001 versus the FA treatment group alone. n = 5. **C-D.** Representative western blot and quantitative data showing the expression of MAGL in different groups. Numbers (1 - 3) indicate each individual animal in a given group. ***P < 0.001 versus the wildtype control group alone; ^###^*P* < 0.001 versus the FA treatment group alone. n = 5.** E.** Representative immunofluorescence micrographs showing renal expression of MAGL in different groups. White arrows indicate positive staining. Scale bar, 50 μm. **F-G.** Representative graphs showing Scr and BUN levels in different groups. *P < 0.05 versus the wildtype control group alone; ^#^*P* < 0.05 versus the FA treatment group alone. n = 5. **H.** Representative graph showing 2-AG levels in kidney homogenates by LC/MS analysis. ***P < 0.001 versus the wildtype control group alone; ^###^*P* < 0.001 versus the FA treatment group alone. n = 5. **I.** Gene expression profiling heatmap by RNA-seq showing differential gene clustering of kidneys from different groups. Numbers (1 - 3) indicate each individual animal in a given group. **J.** Gene ontology (GO) analysis showing differentially expressed genes (DEGs) in 2 groups. Red color indicates the upregulation of biological processes, while blue color indicates the downregulation. **K-N.** Gene set enrichment analysis (GSEA) enrichment plots showing the different enrichment of genes in FA treatment group compared to sham group mice alone or FA/MAGL -CKI group compared to FA treatment group mice alone. *P* value < 0.05. NES: normalized enrichment score. **O-R.** Representative western blot and quantitative data showing the expression of CB2, CB1 and Active-β-catenin in different groups. Numbers (1 - 3) indicate each individual animal in a given group. **P < 0.01, ***P < 0.001 versus the wildtype control group alone; n.s., ^##^*P* < 0.01 versus the FA treatment group alone. n = 5. n.s.: none of significance. **S.** Quantitative data showing renal mRNA levels of CB2 in different groups. ***P < 0.001 versus the wildtype control group alone; ^###^*P* < 0.001 versus the FA treatment group alone. n = 5. **T.** Representative micrographs showing renal expression of CB2 and β-catenin in different groups. White arrows indicate positive staining. Scale bar, 50 μm.

These mice were viable, fertile, and indistinguishable from WT littermates. MAGL knock-in did not affect FAO levels or mitochondrial functions, and no changes in lipid accumulation or fibrosis were observed after MAGL knock-in (Figure S5). FAN model was constructed in MAGL knock-in mice (Figure 6A). The effectiveness of transgene expression was determined by MAGL mRNA and protein levels (Figure 6B-E). Renal function indexes, serum creatinine (Scr), and blood urea nitrogen (BUN), were increased in FAN mice but greatly decreased in MAGL-CKI mice (Figure 6F-G). Notably, MAGL knock-in also reduced 2-AG contents in kidney homogenates (Figure 6H). RNA sequencing showed downregulated fibrosis-related genes, but upregulated FAO-related genes and lipid oxidation in MAGL-CKI mice (Figure 6I-J and Figure S6A). GSEA analysis showed that FA-induced MAGL-CKI mice had inhibited Wnt signaling pathway and extracellular matrix component, and activated FAO and mitochondrial biogenesis compared to FA alone group (Figure 6K-N). We also examined the levels of CB2, CB1, and Active-β-catenin in 3 groups of mice. In FAN mice, CB2 and Active-β-catenin were increased, but their upregulation was blocked by MAGL knock-in. However, CB1 levels remained unchanged after MAGL knock-in (Figure 6O-T).

### Tubular cell specific MAGL knock-in mice resist lipid deposition and renal fibrosis

**Figure 7:**
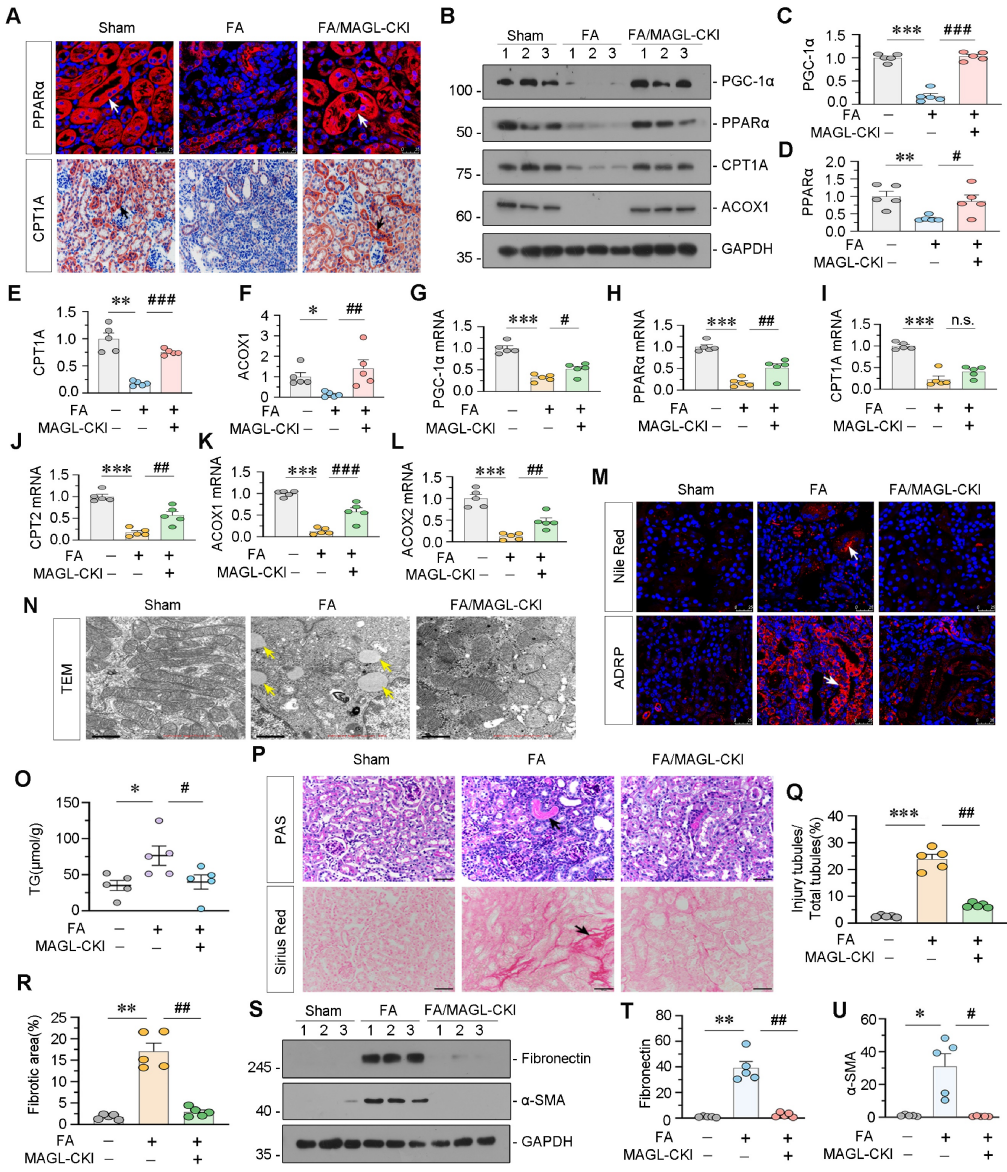
** Tubular cell specific MAGL knock-in mice resist lipid deposition and renal fibrosis in FAN model. A.** Representative micrographs showing the expression of PPARα and CPT1A in kidney. Arrows indicate positive staining. For PPARα staining, scale bar 25 μm; for CPT1A staining, scale bar, 50 μm. **B-F.** Representative western blot and quantitative data showing the expression of PGC-1α, PPARα, CPT1A and ACOX1 in different groups. Numbers (1 - 3) indicate each individual animal in a given group. *P < 0.05, **P < 0.01, ***P < 0.001 versus the wildtype control group alone; ^#^*P* < 0.05, ^##^*P* < 0.01, ^###^*P* < 0.001 versus the FA treatment group alone. n = 5. **G-L.** Quantitative data showing renal mRNA levels of PGC-1α, PPARα, CPT1A, CPT2, ACOX1 and ACOX2 in different groups. ***P < 0.001 versus the wildtype control group alone; n.s., ^#^*P* < 0.05, ^##^*P* < 0.01, ^###^*P* < 0.001 versus the FA treatment group alone. n = 5. n.s.: none of significance. **M.** Representative micrographs showing the expression of lipid (Nile Red) and ADRP in kidney. Arrows indicate positive staining. Scale bar, 25 μm. **N.** Representative TEM images showing lipid droplets in renal tubular epithelial cells in different groups. Yellow arrows indicate lipid droplets. Scale bar, 1 μm.** O.** Representative graph showing renal triglyceride levels in different groups. *P < 0.05 versus the wildtype control group alone; ^#^*P* < 0.05, versus the FA treatment group alone. n = 5. TG: triglyceride. **P.** Representative micrographs showing PAS and Sirius Red staining. Black arrows indicate injury tubules or fibrotic area. Scale bar, 50 μm. **Q-R.** Quantitative analysis of tubular injury and positive staining of fibrotic area in different groups as indicated. Kidney sections were subjected to PAS staining or Sirius Red staining. At least 10 randomly selected fields were evaluated under 400 × magnification and results were averaged for each animal. **P < 0.01, ***P < 0.001 versus the wildtype control group alone; ^##^*P* < 0.01 versus the FA treatment group alone. n = 5. **S-U.** Representative western blot and quantitative data showing the expression of Fibronectin and α-SMA in different groups. Numbers (1 - 3) indicate each individual animal in a given group. *P < 0.05, **P < 0.01 versus the wildtype control group alone; ^#^*P* < 0.05, ^##^*P* < 0.01 versus the FA treatment group alone. n = 5.

Subsequently, we evaluated the protein and mRNA expression levels of FAO-related genes. Consistent with the RNA-seq data, PGC-1α, PPARα, CPT1A, ACOX1, CPT2, and ACOX2 showed decreased expression in FAN mice, while MAGL-CKI increased their expression (Figure 7A-L). We also assessed lipid accumulation in the kidney through diverse experimental methodologies. Nile Red staining and ADRP fluorescence demonstrated substantial lipid deposition in the kidneys of FAN mice, whereas MAGL-CKI ameliorated it (Figure 7M). TEM analysis exhibited augmented lipid droplets in renal tubular cells of FAN mice, but not in MAGL-CKI mice (Figure 7N). Furthermore, the triglyceride (TG) content in kidney tissue was diminished in the MAGL-CKI group (Figure 7O). Periodic acid-schiff (PAS) and Sirius Red staining demonstrated augmented tubular injury and fibrosis in FAN mice, whereas MAGL-CKI mice exhibited diminished levels of these indicators (Figure 7P-R). Likewise, fibrotic proteins such as Fibronectin and α-smooth muscle actin (α-SMA), exhibited increased expression in FAN mice, whereas a substantial decrease was displayed in MAGL-CKI group (Figure 7S-U). Additionally, we assessed the levels of inflammatory factors, confirming that MAGL transgene effectively alleviated renal inflammation (Figure S7A-D).

### Supplement of recombinant MAGL effectively protects against renal fibrosis in UIRI mice

**Figure 8:**
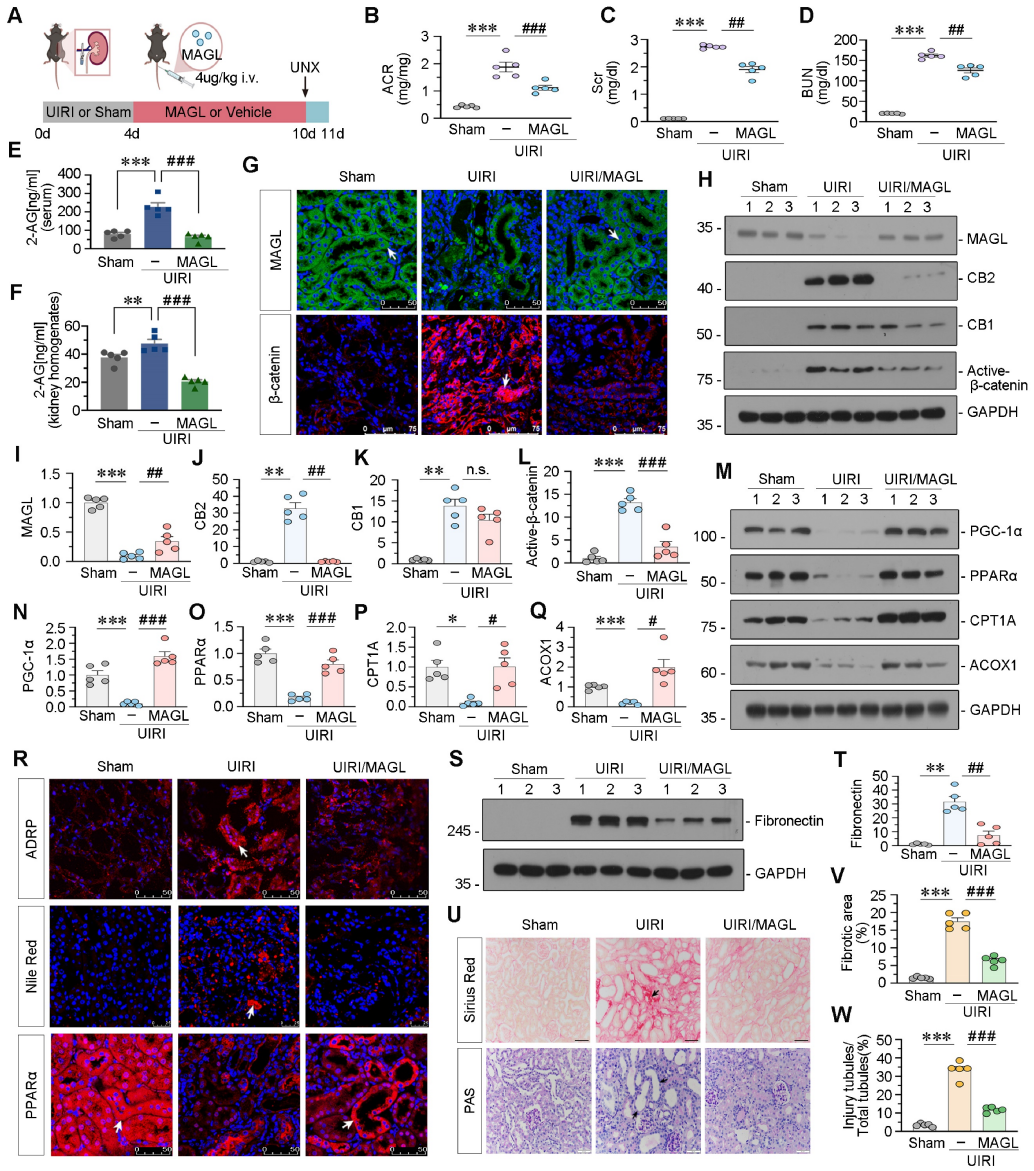
** Supplement of recombinant MAGL effectively protects against renal fibrosis in UIRI mice. A.** Experimental design. Mice were performed UIRI or sham surgery. Red bar indicates intravenous injections of recombinant MAGL protein (4 μg/kg/d) or vehicle. Mice were subjected to unilateral nephrectomy at 10 d and sacrificed at 11 d after UIRI surgery. UNX: unilateral nephrectomy. **B-D.** Representative graphs showing ACR, Scr and BUN levels in different groups. ***P < 0.001 versus the sham control group alone; ^##^*P* < 0.01, ^###^*P* < 0.001 versus the UIRI group alone. n = 5. **E-F.** Representative graphs showing 2-AG levels in serum and kidney homogenates by LC/MS analysis. **P < 0.01, ***P < 0.001 versus the sham control group alone; ^###^*P* < 0.001 versus the UIRI group alone. n = 5. **G.** Representative micrographs showing renal expression of MAGL and β-catenin in different groups. White arrows indicate positive staining. For MAGL staining, scale bar, 50 μm; for β-catenin staining, scale bar, 75 μm. **H-L.** Representative western blot and quantitative data showing the expression of MAGL, CB2, CB1 and Active-β-catenin in different groups. Numbers (1 - 3) indicate each individual animal in a given group. **P < 0.01, ***P < 0.001 versus the sham control group alone; n.s., ^##^*P* < 0.01, ^###^*P* < 0.001 versus the UIRI group alone. n = 5. n.s.: none of significance. **M-Q.** Representative western blot and quantitative data showing the expression of PGC-1α, PPARα, CPT1A and ACOX1 in different groups. Numbers (1 - 3) indicate each individual animal in a given group. *P < 0.05, ***P < 0.001 versus the sham control group alone; ^#^*P* < 0.05, ^###^*P* < 0.001 versus the UIRI group alone. n = 5. **R.** Representative micrographs showing renal expression of ADRP, lipid (Nile Red) and PPARα in different groups. Arrows indicate positive staining. For ADRP and PPARα, scale bar, 50 μm; for Nile Red, scale bar, 25 μm. **S-T.** Representative western blot and quantitative data showing the expression of Fibronectin in different groups. Numbers (1 - 3) indicate each individual animal in a given group. **P < 0.01 versus the sham control group alone; ^##^*P* < 0.01 versus the UIRI group alone. n = 5. **U.** Representative micrographs showing Sirius Red staining and PAS staining. Black arrows indicate fibrotic area or injury tubules. Scale bar, 50 μm. **V-W.** Quantitative analysis of positive staining of fibrotic area and tubular injury in different groups as indicated. Kidney sections were subjected to Sirius Red staining or PAS staining. At least 10 randomly selected fields were evaluated under 400 × magnification and results were averaged for each animal. ***P < 0.001 versus the wildtype control group alone; ^###^*P* < 0.001 versus the FA treatment group alone. n = 5.

Treatment of recombinant MAGL into UIRI mice decreased albumin to creatinine ratio (ACR), Scr, and BUN levels, and reduced 2-AG levels in serum and kidney homogenates (Figure 8A-F). Immunofluorescent staining and western blot analysis demonstrated a reduction in MAGL expression in UIRI mice, concomitant with an elevation in CB2, CB1, and Active β-catenin levels. Nevertheless, the addition of MAGL effectively inhibited the upregulation of CB2 and Active β-catenin, while CB1 levels remained unaltered (Figure 8G-L). FAO-related proteins, were decreased in UIRI mice, but strongly reversed by MAGL treatment (Figure 8M-R). Nile Red staining and ADRP indicated that MAGL supplementation reduced lipid accumulation in UIRI (Figure 8R). Fibronectin, marker of fibrosis, was increased in UIRI but blocked by MAGL treatment (Figure 8S-T). PAS and Sirius Red staining revealed an increase in tubular injury and fibrosis in mice subjected to UIRI. Conversely, the administration of MAGL supplementation retarded tubular injury and fibrosis (Figure 8U-W). Similarly, we examined the inflammatory factors levels, and confirmed that MAGL supplementation also alleviated inflammation (Figure S7E-H).

### 2-AG suppresses PPARα/PGC-1α-mediated FAO via β-catenin signaling

**Figure 9:**
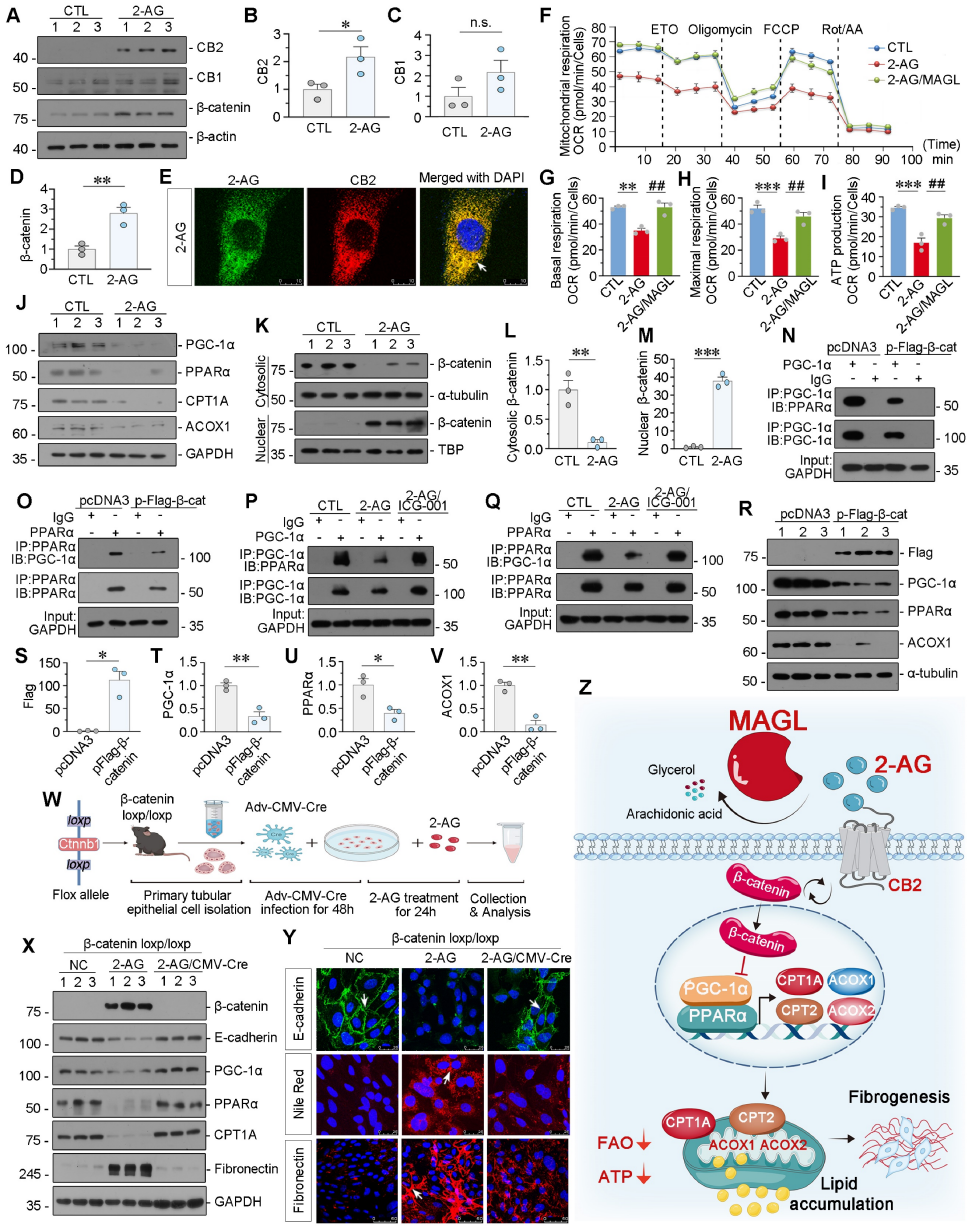
** 2-AG suppresses PPARα/PGC-1α-mediated FAO via β-catenin signaling. A-D.** Representative western blot and quantitative data showing the expression of CB2, CB1 and β-catenin in HK-2 cells treated with 2-AG (100 μM) for 24 h. Numbers (1 - 3) indicate each individual culture in a given group. n.s., *P < 0.05, **P < 0.01 versus the control group alone. n = 3. n.s.: none of significance. **E.** Representative micrographs showing colocalization of CB2 (red) and 2-AG (green) in HK-2 cells. HK-2 cells were treated with 2-AG (100 μM) packaged with indocyanine green material for 2 h. White arrow indicates the colocalization of 2-AG and CB2. Scale bar, 10 μm. **F.** Seahorse analysis of HK-2 cells reveals distinct metabolic profiles across 3 groups: CTL, 2-AG (100 μM), and 2-AG (100 μM) cotreatment with MAGL (100 ng/ml). The cells were treated with etomoxir (ETO), oligomycin, FCCP, rotenone and antimycin (Rot / AA) during the assay. OCR was measured to assess cellular respiration. **G-I**. Quantitative data showing the differences in basal respiration, maximal respiration, and ATP production. **P < 0.01, ***P < 0.001 versus control group alone; ^##^*P* < 0.01 versus 2-AG group alone. n = 3. **J.** Representative western blot showing the expression of PGC-1α, PPARα, CPT1A and ACOX1 in 2 groups. Numbers (1 - 3) indicate each individual culture in a given group. n = 3. **K-M.** Representative western blot and quantitative data showing the expression of β-catenin in cytosolic and nuclear fractions. HK-2 cells were treated with 2-AG (100 μM) for 24 h. The cytosolic and nuclear fractions were isolated and detected by western blot. The expression of cytosolic and nuclear proteins was normalized to α-tubulin and TATA binding protein (TBP) respectively. Numbers (1 - 3) indicate each individual culture in a given group. **P < 0.01, ***P < 0.001 versus control group alone. n = 3. **N-O.** Representative western blot showing β-catenin decreased the binding of PGC-1α and PPARα. HK-2 cells were transfected with β-catenin expression plasmid (pFlag-β-catenin) or pcDNA3.1(+) for 24 h. Cell lysates were immunoprecipitated (IP) with antibodies against PGC-1α or PPARα, followed by immunoblotting (IB) with PPARα or PGC-1α. **P-Q.** Representative western blot showing ICG-001 reversed 2-AG-blocked binding of PGC-1α and PPARα. HK-2 cells were treated with 2-AG (100 μM) alone or treated with ICG-001 (10 μM) for 24 h. Cell lysates were immunoprecipitated (IP) with antibodies against PGC-1α or PPARα, followed by immunoblotting (IB) with PPARα or PGC-1α. **R-V.** Representative western blot and quantitative data showing the expression of Flag, PGC-1α, PPARα and ACOX1 in HK-2 cells transfected with β-catenin expression plasmid (pFlag-β-catenin) or pcDNA3.1(+) for 24 h. Numbers (1 - 3) indicate each individual culture in a given group. *P < 0.05, **P < 0.01 versus the pcDNA3.1(+) group alone. n = 3. **W.** Experimental design. Primary tubular epithelial cells were isolated from β-catenin loxp/loxp mice and transfected with Adv-CMV-Cre virus to knock out β-catenin and then treated with 2-AG (100 μM) for 24 h. **X.** Representative western blot showing the expression of β-catenin, E-cadherin, PGC-1α, PPARα, CPT1A and Fibronectin in different groups. Numbers (1 - 3) indicate each individual culture in a given group. n = 3. **Y.** Representative immunofluorescence micrographs showing the expression of E-cadherin, lipid (Nile Red) and Fibronectin in different groups. White arrows indicate positive staining. For E-cadherin and Nile Red staining, scale bar, 25 μm; for Fibronectin staining, scale bar, 50 μm. **Z.** The schematic diagram demonstrates 2-AG binds to the receptor CB2 to induce β-catenin activation and then inhibits transcriptional activity of PGC-1α/PPARα to block FAO-related genes expression, which results in lipid accumulation and subsequent fibrogenesis. Of note, MAGL could hydrolyze 2-AG to block the whole pathway, and then ameliorates lipid toxicity and fibrogenesis in renal tubular cells.

We then treated HK-2 cells with 2-AG. 2-AG upregulated CB2 and β-catenin but did not affect CB1 (Figure 9A-D). 2-AG was then packaged with ICG material to be visual. Of interest, we observed co-staining of 2-AG and CB2, further suggested 2-AG induced cell injury through CB2 (Figure 9E). Mitochondrial oxygen consumption rate (OCR) was measured by Seahorse analysis. Cellular OCR was determined in the basal state and upon exposure to oligomycin, p-trifluoromethoxy carbonyl cyanide phenylhydrazine (FCCP), rotenone and antimycin (Rot/AA). Etomoxir (ETO), a selective inhibitor of CPT-1, was also added to evaluate the extent of FAO. The Seahorse analysis revealed a reduction in basal and maximal OCR, as well as ATP production capacity in the cells treated with 2-AG group. Whereas, supplementation with MAGL restored these parameters (Figure 9F-I). These findings indicate that MAGL effectively restores mitochondrial FAO metabolism. In addition, 2-AG significantly reduced the protein levels of FAO-related genes in vitro (Figure 9J and Figure S8A-D). We then isolated cytosolic and nuclear proteins. 2-AG treatment induced nuclear translocation of β-catenin, the active form of β-catenin (Figure 9K-M). Immunoprecipitation analysis showed β-catenin overexpression decreased the binding of PGC-1α and PPARα (Figure 9N-O). Pre-treatment with the small molecule compound ICG-001 (inhibitor of β-catenin) reversed 2-AG-decreased PGC-1α binding with PPARα binding (Figure 9P-Q). Simultaneously, β-catenin decreased the expression of PGC-1α, PPARα, and ACOX1 (Figure 9R-V). To further confirm the role of β-catenin, tubular cells were isolated from β-catenin loxp/loxp mice and treated with 2-AG following the induction of β-catenin knockout (Figure S8E-G and Figure 9W). 2-AG downregulated the expression of E-cadherin, PGC-1α, PPARα, and CPT1A in primary cells, while inducing upregulation of β-catenin, Fibronectin, and lipid accumulation. However, these effects were effectively blocked by β-catenin knockout (Figure 9X-Y and Figure S8H-M). These findings suggest β-catenin plays a crucial role in 2-AG-induced lipid metabolism dysfunction and renal fibrosis.

Taken together, the schematic diagram demonstrates 2-AG binds to the receptor CB2 to induce β-catenin activation, and then inhibits transcriptional activity of PGC-1α and PPARα to block FAO-related genes expression, which results in lipid accumulation and subsequent fibrogenesis. Of note, MAGL could hydrolyze 2-AG to block the whole pathway, and then ameliorate lipid accumulation and fibrogenesis. MAGL is a promising therapeutic strategy for CKD treatments (Figure 9Z).

## Discussion

CKD affects around 159 million people in China, with 26.4 million suffering from end-stage renal disease (ESRD) 39. As to no efficacious therapies, CKD is becoming a significant public health problem worldwide. To early diagnose and effectively intervene is of utmost importance to nephrologists.

Tubular cells are responsible for absorption and secretion in kidney, and require large amounts of energy to remain active and survive 40. Energy metabolism dysfunction in tubular cells plays a key role in triggering CKD 41. However, the underlying mechanisms and regulatory strategies have not been fully evaluated.

FAO is a process of fatty acid metabolism regulated by PPARα 42,43, which controls important genes like CPT1, CPT2, ACOX1, and ACOX2 44. PGC-1α enhances PPARα activity 45. FAO dysfunction causes fatty acid accumulation, leading to tubular injury and fibrosis 23,46. Enhancing PPARα activity preserves FAO function, inhibits tubular injury, and slows fibrosis 47,48. However, further investigation is needed to identify PGC-1α and PPARα regulators.

ECS is an important system in the body, comprising endocannabinoids, their receptors, and regulatory enzymes. ECS is highly involved in several physiological functions, including appetite, immune regulation, pain regulation, inflammation modulation, metabolic, and also in kidney diseases 49,50. Previous studies found CB2 triggers renal fibrosis 31,33 and may be involved in energy metabolism and lipid metabolism in renal tubular cells. However, the detailed mechanism has not been understood. Studies have shown 2-AG is a potent activator of CB2 receptor 51,52. In UUO mice, 2-AG levels increased in kidneys 34. However, whether 2-AG can directly cause tubular cell injury and lipid metabolism dysfunction has not been investigated.

In this study, we found serum 2-AG levels increased significantly in CKD (Figure 1). Moreover, we observed 2-AG is enriched in injured kidney of UUO. And 2-AG activated CB2/β-catenin pathway to accelerate renal lipid accumulation and fibrosis progression (Figure 2 and Figure S1). Mechanistically, 2-AG induced CB2 and β-catenin activation, to inhibit PGC-1α/PPARα-mediated FAO function (Figure 9). These results suggest 2-AG induces FAO dysfunction and lipid accumulation through CB2/β-catenin pathway.

MAGL is an enzyme that regulates the levels of 2-AG in the body. It was initially discovered in the intestines and adipose tissues of rats and consists of an alpha/beta hydrolase fold and a catalytic triad 53. MAGL plays a crucial role in regulating the endocannabinoid system, and blocking MAGL in the nervous system can increase 2-AG levels and activate the 2-AG signaling 54. MAGL also indirectly affects the levels of free fatty acids and controls the levels of other lipids with pro-inflammatory or tumor-promoting effects through their hydrolysis 55. Inhibition of MAGL has been shown to enhance 2-AG-mediated CB2 signaling and ameliorate injury and inflammation caused by hepatic ischemia-reperfusion 56. MAGL inhibition can exacerbate acute myocardial infarction in mice by mobilizing myeloid cells through 2-AG 57. Increased MAGL expression promotes cancer cell invasion and tumor growth by regulating free fatty acid levels in human cancers 58. Besides regulating ECS, MAGL is also involved in various other processes such as lipid catabolism, energy metabolism, and activation of lipid-related signal transduction pathways 36,59. However, the role of MAGL in kidney diseases has not been well understood.

Interestingly, we observed a decrease in MAGL expression in kidneys of both multiple mice models and clinical patients (Figure 3). We also ascertained the possibility of MAGL being the potential prognostic factor in CKD (Figure 4). We therefore speculated that MAGL can be applied as a strategy to prevent and treat 2-AG-induced fibrosis. The role of MAGL was comprehensively assessed by genetic and pharmacological approaches in CKD models. The efficacies of MAGL to improve renal function and ameliorate kidney fibrosis were confirmed by studies in transgenic mice, exogenous application of recombinant protein (Figure 5-8). In contrast to its promoting role of MAGL in liver injury, our findings for the first time indicate MAGL protects against lipotoxicity and inflammation in renal fibrosis (Figures 5-8, S7). This implies that MAGL may exert distinct effects in the liver and kidneys, possibly due to tissue-specific and cell-specific actions.

Our new findings show MAGL, besides serving as a monoacylglycerol lipase involving direct lipid hydrolysis, innovatively exhibits special roles in FAO metabolism related to 2-AG/CB2 signaling. This dual functionality effectively alleviates lipotoxicity in renal tubular cells. Consequently, MAGL emerges as a promising candidate for mitigating tubular cell damage and renal fibrosis. Large quantities of researches have confirmed the substantial accumulation of triglycerides and saturated free fatty acids in CKD, both of which contribute to organelle structural damage, oxidative stress, inflammatory responses, and cell death. Various pharmacological interventions, such as lipid-lowering drugs of fluvastatin 60, PPARα agonist fenofibrate 61, and the sodium-glucose cotransporter 2 (SGLT2) inhibitor canagliflozin 62, could greatly attenuate lipid-induced kidney injury and fibrosis. In contrast, MAGL, being an endogenously well-expressed component in the kidneys, presents a significant advantage compared to those small compounds. Replenishing MAGL during renal injury demonstrates its dual efficiencies in lipid hydrolysis and FAO, making it a promising avenue for further investigation and therapeutic intervention study.

β-catenin is normally silent in adult kidneys but becomes reactivated in CKD and renal fibrosis 63. β-catenin could induce multiple targets to trigger tubular cell injury 64-66. We found β-catenin not only mediates CB2-induced renal tubular injury but also inhibits PGC-1α 29,33, an important regulator for FAO function. To investigate if β-catenin mediates 2-AG-induced lipid metabolism dysfunction, we isolated primary cultured tubular cells from β-catenin-knockout and used inhibitor methods to find that β-catenin suppresses PGC-1α and PPARα and impedes their interaction, ultimately leading to impaired FAO function (Figure 9).

Therefore, we innovatively discovered that MAGL, through hydrolyzing 2-AG, effectively restores FAO by suppressing 2-AG/β-catenin signaling, thereby alleviating lipotoxicity in tubular cells and mitigating renal fibrosis. Our study revealed a novel mechanism underlying lipid accumulation in renal tubular cells during CKD. Importantly, our findings propose that MAGL not only holds promise as a diagnostic indicator for renal function decline but also emerges as a new therapeutic target for CKD.

### Concise methods

For detailed methods, see the Supplementary Methods.

### Human clinical specimens

Human specimens (urine, serum, and kidney biopsies) were collected from CKD patients at the First People's Hospital of Foshan. Normal control biopsies were obtained from paracancerous tissues of patients undergoing nephrectomy. Demographic and clinical data are presented in Supplementary Table S1 - S3. Studies involving human samples were approved by the Medical Ethics Committee of the First People's Hospital of Foshan (FSYYY - EC - SOP - 008 - 02.0 - A09) and performed with informed patient consent.

### MAGL enzyme-linked immunosorbent assay

We used an ELISA kit to measure urinary MAGL concentration and corrected it by urine creatinine.

### Animal models

Male C57BL/6 mice were purchased from the Experimental Animal Center of Southern Medical University. Tubule-specific MAGL conditional knock-in mice were purchased from Cyagen Biosciences.

We used different methods to induce various kidney disease models in mice: unilateral ureteral obstruction (UUO) by double-ligation of the left ureter, folic acid-induced nephropathy by intraperitoneal injection, unilateral ischemia reperfusion injury (UIRI) by renal pedicle clipping.

Animal studies were performed according to the Guidelines for the Care and Use of Laboratory Animals and approved by the Animal Ethics Committee at Nanfang Hospital, Southern Medical University (NFYY - 2020 - 0837).

### Generation of β‐catenin loxp/loxp mice

C57BL/6 β‐catenin loxp/loxp mice were generated by CRISPR/Cas9 system.

### Tubule-specific MAGL conditional knock-in mice and genotyping

The construction of MAGL-CKI was achieved by applying CRISPR/Cas9 for the knock-in of MAGL gene into Rosa26 of C57BL/6 zygotes.

### Urinary albumin, serum creatinine and BUN assay

Urinary albumin was measured using a mouse Albumin ELISA Quantitation kit, and standardized to urine creatinine. Serum creatinine and BUN levels were determined by an automatic chemistry analyzer.

### Preparation of 2-AG Nanoparticles

We prepared liposomes containing DPPC: DSPE-PEG2000 in a 95 : 5 molar ratio using the thin film hydration method^53^. Lipid and 2-AG in a weight ratio of 20 : 1 were dissolved in 30 ml CHCl3 and evaporated at 25 °C to form a thin lipid film. Residual solvent was removed in vacuum for 6 h. ICG in a PBS solution (500 μg/mL) was added to the lipid film and encapsulated by rotary evaporation at 25 °C. The crude liposome was extruded 11 times through a 100 nm filter using an Avanti Polar Lipids mini-extruder.

### *In vivo* Bioimaging of 2-AG distribution

After Sham or UUO surgery, C57BL/6 mice were intravenously injected with 2-AG-loaded nanoparticles at 10 mg/kg body weight, 3 d after surgery under general anesthesia. 2 h later, fluorescence images were visualized using a Bruker FX PRO imaging system with excitation at 785 nm and emission at 810 nm. All procedures were conducted in the dark.

### LC-MS

To prepare plasma, cell, or kidney tissue samples, we added 200 μl of toluene to 200 μl of plasma, 10^7 cells, or 20 mg of kidney tissue. After grinding, crushing, and centrifuging at 13,000 rpm at 4 ℃ for 10 min, we transferred the upper organic phase to a 1.5 ml EP tube and dried it using a nitrogen blower. The residue was then resolved by adding 100 μl of 75% methanol and shaking for 30 s. LC-MS analysis was performed after centrifuging at 14,000 rpm at 4 ℃ for 20 min. We used LC-MS grade solvents and the following standard reagents: 2-AG and AEA.

### Transmission Electron Microscopy

Kidney cortex was fixed in 1.25% glutaraldehyde/0.1 M phosphate buffer. Ultrathin sections (60 nm) were prepared by a routine procedure and examined under an electron microscope.

### Western blot analysis and immunoprecipitation

Protein expression was analyzed by western blot analysis. The interaction among proteins was detected by coimmunoprecipitation 67. Primary antibodies used were described in the Supplementary Methods.

### Reverse transcription and real‐time PCR

RNA was isolated using TRIzol RNA isolation system, and real-time PCR was performed on an ABI PRISM 7000 Sequence Detection System. The primer sequences are listed in Supplementary Table S4.

### Cell Culture and Treatment

Human proximal tubular cell line (HK-2) was purchased from the Cell Bank of the Chinese Academy of Sciences. Nuclear and cytoplasmic fractions were separated with a commercial kit. ATP production was determined using enhanced ATP assay kit.

### Seahorse assay

HK-2 cells were treated with 2-AG alone or in combination with MAGL, and their metabolic profiles were assessed using a Seahorse XF96 Analyzer. OCR was measured, and mitochondrial function and FAO were evaluated through specific injections.

### Isolation of tubular epithelial cells and treatment

We isolated and cultured primary mouse kidney tubular cells from β-catenin loxp/loxp mice or MAGL-CKI mice following a routine protocol, which involved mincing the kidneys and digesting them with collagenase. After growing the cells for 4-8 d, the cells isolated from β-catenin loxp/loxp mice were transfected with Adv-CMV-Cre and then treated with 100 μM 2-AG for 24 h. The primary renal tubular epithelial cells isolated from MAGL-CKI mice were treated with TGF-β1 (5 ng/ml) for 24 h. We then harvested the cells for analysis.

### Histology, immunohistochemical and immunofluorescence staining

Paraffin-embedded kidney sections were performed with Periodic Acid-Schiff (PAS) and Sirius Red staining to identify injured tubules and collagen deposition. Nile Red staining, immunohistochemical and immunofluorescence staining were performed using routine protocols. Antibodies are described in the Supplementary Methods.

### Transcriptomic analysis

RNA-seq was performed on kidney tissues from different groups of mice using TRIzol reagent and Illumina Novaseq platform. Gene expression was quantified, and differential expression analysis was conducted with DESeq2. Pathway enrichment analysis was performed using clusterProfiler and Reactome, while GSEA analysis was conducted using various databases.

### Untargeted Metabolomics

Tissue samples were collected and prepared according to the manufacturer's instructions. UHPLC-MS/MS analyses were completed by an UHPLC (ThermoFisher, Germany) coupled with an Orbitrap Q ExactiveTM HF mass spectrometer (Thermo Fisher, Germany) in Novogene Co., Ltd. (Beijing, China). The raw data were processed by the Compound Discoverer 3.3 (CD3.3, ThermoFisher), including peak alignment, peak picking, and metabolite identification. Pareto-scaled principal component analysis (PCA) and orthogonal partial least-squares discriminant analysis (OPLS - DA) were performed at meta X. The metabolites with Variable Importance in the Projection (VIP) > 1 and *P*-value < 0.05 were assigned as significant changed.

### Statistical analyses

Data were presented as mean with SEM, and statistical analysis was performed using IBM SPSS Statistics 25. We used Chi-square test for comparing two rates or two composition ratios. For the parametric analysis, we used Student's t-test for comparing two groups and one-way ANOVA followed by the Least Significant Difference or Dunnett's T3 procedure for comparing more than two groups. A *P*-value < 0.05 was considered significant. Bivariate correlation analysis was conducted using Pearson and Spearman rank correlation analysis.

### Data availability

Transcriptomics data in this study has been uploaded to the NCBI SRA database (accession number: PRJNA987376, PRJNA974908). The metabolomics data in this study are available in the MetaboLights database (MTBLS9333). The authors are willing to provide other raw data that support the findings of this article to qualified researchers without any hesitation.

## Supplementary Material

Supplementary methods, figures and tables.
