# Supplementary material for: MAGL protects against renal fibrosis through inhibiting tubular cell lipotoxicity
Source: Theranostics. 2024 Feb 4;14(4):1583–601. doi: 10.7150/thno.92848 (PMC10879875; doi:10.7150/thno.92848)
Supplement: Supplementary file 1 — Supplementary methods, figures and tables. [file thnov14p1583s1.pdf]

Supplementary Materials for

MAGL protects against renal fibrosis through inhibiting tubular cell lipotoxicity

Shan Zhou[1,6], Xian Ling[1,6], Jielin Zhu[1,2,6], Ye Liang[1,6], Qijian Feng[1,6], Chao Xie[3,6], Jiemei Li[1], Qiyan Chen[1,3], Shuangqin Chen[4], Jinhua Miao[1], Mengyao Zhang[1], Zhiru Li[1], Weiwei Shen[1], Xiaolong Li[1], Qinyu Wu[1], Xiaoxu Wang[1], Ruiyuan Liu[5], Cheng Wang[4], Fan Fan Hou[1], Yaozhong Kong[3]*, Youhua Liu[1]*, Lili Zhou[1]*

Correspondence to: jinli730@smu.edu.cn

This file includes:

Supplementary Detail Methods

Supplementary Figure S1 to S8

Supplementary Table S1 to S4

Supplementary Detail Methods

Human clinical specimens

All human specimens (urine, serum and kidney biopsies) were collected from patients with CKD at the First People's Hospital of Foshan. CKD was diagnosed based on an estimated glomerular filtration rate (eGFR). 24 h urine and serum samples were collected in cryotubes respectively and stored at -80 °C. Normal control biopsies were obtained from paracancerous tissues of patients who had renal cell carcinoma and underwent nephrectomy. The demographic and clinical data are presented in Supplementary Table. All the studies involving human samples were performed with informed patient consent and approved by the Medical Ethics Committee of the First People's Hospital of Foshan (FSYYY – EC – SOP – 008 - 02.0 - A09).

MAGL enzyme-linked immunosorbent assay

The urinary concentration of MAGL was analyzed by human Monoglyceride lipase ELISA kit (CSB - EL013787HU; CUSABIO Life Science, Wuhan, China) and corrected by urine creatinine.

Animal models

Male C57BL/6 mice weighing 20 – 22 g were purchased from the Experimental Animal Center of Southern Medical University (Guangzhou, China). Tubule-specific MAGL conditional knock-in mice were purchased from Cyagen Biosciences. All mice undergoing surgeries were treated with general anesthesia and housed in Experimental Animal Center of Southern Medical University under pathogen-free conditions.

For studying the effects of 2-AG, the UUO model was established by triple-ligating the left ureter with 4-0 silk after an abdominal midline incision. At 3 d after operation, mice were subjected to daily intravenous injections of 2-AG Nanoparticles for 7 d in the dark room. Mice were sacrificed at 10 d

after UUO surgery. Mice have randomly divided into 4 groups: (i) sham controls; (ii) mice treated with 2-AG; (iii) UUO mice treated with vehicle; (iv) UUO mice treated with 2-AG.

For the folic acid–induced nephropathy model, mice were administered a single intraperitoneal injection of folic acid at 250 mg/kg body weight. Tubule-specific MAGL conditional knock-in mice and matched C57BL/6 mice were sacrificed at 10 d after FA injection.

For the UIRI model, the left renal pedicle was clipped for 35 min using microaneurysm clamps. At 4 d after operation, mice were daily injected intravenously with Recombinant Human MAGL Protein (Catalog: 7930-MG; R&D Systems) at 4 μg/kg through the tail vein for a week by a hydrodynamic-based gene delivery approach. After 10 d post-IRI, the right kidney was removed via a right flank incision. Mice were sacrificed at 11 d post-IRI, respectively. Mice have randomly divided into 3 groups: (i) sham controls; (ii) UIRI mice treated with vehicle; (iii) UIRI mice treated with Recombinant Human MAGL Protein.

The detailed experimental designs were shown in Figures 2A, 6A, 8A.

For ADR model, male BALB/c mice were treated with ADR via a single intravenous injection of 11 mg/kg body weight. Mice were killed at 1, 3 and 5 w after ADR injection.

All mice were randomly divided into different groups as indicated, using the online tool “Research Randomizer” (https://www.randomizer.org). 5 mice were included in each group. All animal studies were performed in accordance with the Guidelines for the Care and Use of Laboratory Animal and approved by the Animal Ethics Committee at the Nan fang Hospital, Southern Medical University (NFYY-2020- 0837).

Generation of β-catenin loxp/loxp mice

The β-catenin loxp/loxp mice were generated in C57BL/6 background by CRISPR/Cas9 system and were purchased from Cyagen Biosciences (stock no. CKOCMP - 12387 - Ctnnb1 - B6N - VA; Cyagen Biosciences, Guangzhou, China). The genotyping of tail DNA samples was confirmed by RT-PCR.

Tubule-specific MAGL conditional knock-in mice and genotyping

The construction of MAGL-CKI was achieved by applying CRISPR/Cas9 for the knock-in of MAGL gene into Rosa26 of C57BL/6 zygotes. Cdh16-cre recombinase removed the stop signal (a translation interrupting Loxp-Stop-Loxp cassette) between the loxP sites. A high level of MAGL expression was driven by the CAG promoter.

The MAGL-CKI transgenic mice were purchased from Cyagen Biosciences (TIS190827MG1; Cyagen Biosciences, Guangzhou, China). The genotyping of tail DNA samples was confirmed by RT-PCR.

Urinary albumin, serum creatinine and BUN assay

Serum creatinine and BUN levels were determined by an automatic chemistry analyzer (AU480 Chemistry Analyzer, Beckman Coulter, Atlanta, Georgia). The data were expressed as mg/dl. Urinary albumin was measured using a mouse Albumin ELISA Quantitation kit (Bethyl Laboratories, Montgomery, TX), and standardized to urine creatinine.

Preparation of 2-AG Nanoparticles

Liposome containing DPPC : DSPE-PEG2000 = 95:5 (molar ratio) was prepared by the reported thin film hydration method[1]. Normally, lipid: 2-AG = 20 : 1 (weight ratio) was fully dissolved in 30 ml

CHCl3, and CHCl3 was removed by rotary evaporation at 25 °C until a thin lipid film was formed. Residual solvent in liposome was removed in vacuum for 6 h. A PBS solution of indocyanine green (ICG) (500 μg/ml, Apexbio) was added to the lipid film, and ICG was encapsulated by rotary evaporation at 25 °C. The crude liposome was extruded through a 100 nm filter for 11 times using an Avanti Polar Lipids mini-extruder (Alabaster, AL).

***In vivo* Bioimaging of 2-AG distribution**

C57BL/6 mice were subjected to sham or UUO surgery. 3 d after surgery, mice were intravenously injected with 2-AG-loaded nanoparticles at 10 mg/kg body weight after general anesthesia. 2 h later, the anesthetized mice were placed into the chamber, and the fluorescence images were visualized using a Bruker FX PRO imaging system equipped with an excitation at 785 nm and emission at 810 nm. All procedures were conducted in dark.

Nuclear and cytoplasmic fraction isolation

Nuclear and cytoplasmic fractions were separated with a commercial kit (BB-3102; BestBio, Shanghai, China) according to the manufacturer's protocol.

Isolation of tubular epithelial cells and treatment

Primary mouse kidney tubular epithelial cells were isolated and cultured using routine protocol. Briefly, the kidneys of β-catenin loxp/loxp mice or tubule-specific MAGL conditional knock-in mice were peeled off and minced, then digested with 0.75 mg/ml collagenase (Cat No 4188; Worthington) for 25 min at 37 °C.

The tubular tissues were isolated by 100 μm cell filter and then they were centrifuged using 31% Percoll gradients, resuspended and washed twice with DMEM/F-12. Finally, tubules were suspended in DMEM/F-12 supplemented with 10% bovine calf serum, 50 U/ml penicillin and 50 mg/ml streptomycin. Cells were grown in cell culture dishes for 4-8 d until they reached about 60% confluency.

The primary renal tubular epithelial cells isolated from β-catenin loxp/loxp mice were transfected with Adv-CMV-Cre (GCD0320409; Genechem Shanghai, China) for 48 h according to the manufacturer's protocol. Then 2-AG at 100 μM was added into the cells for another 24 h.

The primary renal tubular epithelial cells isolated from tubule-specific MAGL conditional knock-in mice were treated with TGF-β1 (5 ng/ml) for 24 h.

The cells were harvested for immunofluorescence and protein analyses.

Cell Culture and Treatment

HK-2 was purchased from the Cell Bank of the Chinese Academy of Sciences (Shanghai, China) and cultured in DMEM/F12 medium (Biological Industries) supplemented with 10% fetal bovine serum (Biological Industries). No contamination was detected.

HK-2 cells were synchronized into quiescence by growing in serum-free medium, and then treated with 2-AG (100 μM, Catalog No. 1298; Tocris Bioscience) for 24 h. Some cells were pretreated with recombinant MAGL protein (100 ng/ml, Catalog No. 7930-MG; R&D) for 1 h, and then treated with TGF-β1 (5 ng/ml, Catalog No. 7754-BH; R&D) for 24 h. In some experiments, HK-2 cells were treated with TGF-β1 (5 ng/ml) alone or cotreated with JZL-184 (20 μmol/ml, ab141592; Abcam) for 24 h.

Seahorse assay

HK-2 cells (50,000 cells/well) were seeded in Seahorse XF96 cell culture microplates and subjected to various treatments (2-AG alone or in combination with MAGL). Following a 1-hour incubation in Seahorse XF DMEM medium supplemented with 10 mM XF glucose solution, 1 mM XF sodium pyruvate solution, and 2 mM glutamine solution, baseline measurements of basal OCR were recorded using a Seahorse XF96 Analyzer. To assess mitochondrial function, sequential injections of oligomycin (1.5 μM), FCCP (1 μM), rotenone and antimycin (0.5 μM) were administered, providing insights into maximal OCR and mitochondrial respiratory parameters. To evaluate FAO, Etomoxir (4 μM) was introduced. All reagents were provided by Agilent Technologies as part of the Seahorse XF Cell Mito Stress Test Kit (Catalog Number: 103674 - 100). The collected data were meticulously analyzed to elucidate cellular metabolism and energy production, offering a comprehensive understanding of metabolic dynamics under different experimental conditions.

Western blot analysis

Protein expression was analyzed by western blot analysis. Briefly, total proteins were extracted from renal tissues and cell pellets with lysis buffer. Protein concentration were measured using BCA protein concentration determination. Proteins were subjected to SDS-PAGE electrophoresis and transferred to polyvinylidene difluoride (PVDF) membrane (Merck Millipore Ltd, IPVH00010, Ireland), then blocked in 5% of milk and incubated with different primary antibodies at 4 °C overnight. The next day, PVDF membrane was incubated with a responding secondary antibody for 1 h at room temperature, and visualized by an ECL kit (Applygen, Beijing, China). The primary antibodies used were as follows: anti-CB2 (ab45942; Abcam), anti-Fibronectin (F3648; Sigma), anti-α-SMA (a2547; Sigma-Aldrich), anti-α-SMA (ab5694; Abcam), anti-β-catenin (610154; BD Biosciences), anti-PGC-1α (ab54481;

Abcam), anti-TOMM20 (ab186735; Abcam), anti-α-tubulin (RM2007; Ray Antibody Biotech), anti-GAPDH (RM2002; Ray Antibody Biotech), anti-β-actin (RM2001; Ray Antibody Biotech), anti-Collagen I (BA0325; Boster, Biotechnology), anti-CB1 (BA2144; Boster Biotechnology), anti-Active-β-catenin (4270s; Cell Signaling Technology), anti-CPT1A (ab128568; Abcam), anti-ACOX1 (A8091; ABclonal), anti-PPARα (A18252; ABclonal), anti-Flag-tag (M185-3; MBL), anti-TBP (ab818; Abcam), anti-MAGL (ab228598; Abcam), anti-E-cadherin (ab76055; Abcam), anti-Vimentin (5741s; Cell Signaling Technology).

Immunoprecipitation

The protein-protein interaction was assessed by co-immunoprecipitation as previously described[2]. HK-2 cells were pretreated with ICG-001 (10 μM) before treated with 2-AG (100 μM) or transfected with β-catenin expression plasmid (pFlag-β-catenin) alone. Cell lysates were immunoprecipitated with antibodies against anti-PPARα (A18252; ABclonal) or anti-PGC-1α (ab54481; Abcam), followed by immunoblotting with anti-PPARα (A18252; ABclonal), and anti-PGC-1α (ab54481; Abcam).

Triglycerides (TG) assay

Triglycerides was tested using a commercial kit (E1025-105; APPLYGEN) according to the manufacturer's protocol.

Nile Red Staining

Kidney cryosections or HK-2 cells cultured on coverslips were fixed with 4% paraformaldehyde for 10 min at room temperature, followed by washing with PBS. After permeabilizing with 0.1% Tween 20

for 5 min, the cells or frozen sections were incubated with a Nile red (1 μg/ml, 72485; Sigma) and DAPI (Sigma-Aldrich) dual staining solution for 10 min in dark. Images were taken by confocal microscopy (Leica TCS SP2 AOBS, Leica Microsystems, Buffalo Grove, IL).

Immunofluorescence staining

Kidney cryosections were fixed with 4% paraformalin fixing solution for 15 min at room temperature. HK-2 cells cultured on coverslips were fixed with cold methanol: acetone (1 : 1) for 15 min at room temperature, followed by blocking with 10% normal donkey serum in PBS. Slides were incubated with primary antibodies against anti-CB2 (ab45942; Abcam), anti-CB2 (sc-293188; santa cruz), anti-Fibronectin (F3648; Sigma), anti-α-SMA (ab5694; Abcam), anti-β-catenin (610154; BD Biosciences), anti-A ctive-β-catenin (4270s; Cell Signaling Technology), anti-PPARα (A18252; ABclonal), anti-MAGL (ab228598; Abcam), anti-MAGL (sc-398942; santa cruz), anti-ADRP (ab52356; Abcam), anti-NCC (AB3553; Sigma-Aldrich), anti-AQP3 (ab125219; Abcam), anti-AQP1 (ab9566; Abcam), anti-E-cadherin (ab76055; Abcam), anti-Lotus Tetragonolobus Lectin (LTL) (FL-1321; VECTOR Laboratories), anti-Peanut Agglutinin (PNA) (FL-1071; VECTOR Laboratories), and anti-Dolichos Biflorus Agglutinin (DBA) (FL1031; VECTOR Laboratories). After washing with TBS-T, slides were incubated with Cy2 or Cy3-conjugated donkey anti-mouse or anti-rabbit IgG (Jackson Immuno-Research Laboratories, West Grove, PA). Nuclei were stained with DAPI (Cat. C1006; Beyotime) according to the manufacturer's instructions. Images were captured using confocal microscopy (Leica TCS SP2 AOBS; Leica Microsystems, Buffalo Grove, IL).

Histology and immunohistochemical staining

Paraffin-embedded (3 μm) mouse kidney sections were prepared using routine protocols. Sections were stained with Sirius red staining to identify collagen deposition. Some sections were stained with periodic acid-Schiff (PAS) (BA4080A; BASO). Immunohistochemical staining was performed using routine protocol. The primary antibodies used were as follows: anti-CPT1A (ab128568; Abcam), anti-fibronectin (F3648; Sigma), anti-β-catenin (610154; BD Biosciences) and anti-MAGL (ab228598; Abcam). Images were taken by a microscope DP 27 CCD camera (Olympus, Japan).

Quantifications of staining

Slides stained with Sirius red, immunohistochemical and immunofluorescence were observed at high magnification (x 400, x 1000) fields from randomly selected fields. Each section contained 10 fields, and the image of each part was divided into 100 squares. The tissue fibrosis stained in red was scored. Quantification of fibrotic lesions or positive area was assessed by the Image Pro plus software V6.0 (Media Cybernetics, Inc., Rockville, USA). The injury score was assessed by tubular dilation, hyaline casts and detached epithelial cells in tubular lumens as well as detached brush borders. The percentages of tubular injury in each image were calculated by three experienced observers in a blinded fashion.

LC-MS

Plasma pretreatment was conducted by melting the frozen samples on an ice surface and absorbing 200 μl plasma. 10^7 cells were digested by pancreatic enzyme and then centrifuged at 4 °C and 800 rpm for 5 min. 200 μl of toluene was added to the plasma and cell precipitates samples and the mixture were centrifuged for 10 min at 13000 rpm at 4 °C for 30 s after shock. The kidney tissue samples (weighing 20 mg) were pretreated by adding 200 μl of toluene. The mixture was ground, crushed, and

then centrifuged at 13000 rpm at 4 °C for 10 min after shaking for 30 s. The upper organic phase was then transferred to a 1.5 ml EP tube and dried using a nitrogen blower. The residue was resolved by adding 200 μl of 75% methanol and vortexed for 30 s. The sample was then centrifuged at 14000 rpm at 4 °C for 20 min and subjected to LC-MS analysis. For all liquid chromatography-mass spectrometry (LC-MS) methods, LC-MS grade solvents were used. The standard reagents used were as follows: 2-AG (Item No.62160; Cayman Chemical); AEA (Catalog No.1339; Tocris Bioscience).

Transmission Electron Microscopy

Kidney cortex and HK-2 cells were fixed in 1.25% glutaraldehyde/0.1 M phosphate buffer, followed by resin embedding and ultrafine section making. Slides were subjected to assess kidney tubular mitochondrial ultrastructure and lipid droplets under an electron microscope (JEOL JEM - 1010, Tokyo, Japan).

ATP assay

ATP concentrations of HK-2 cells were assessed by enhanced ATP assay kit (S0027; Beyotime Biotechnology), according to the manufacturer's protocol.

Reverse transcription (RT) and real-time PCR

Total RNA was obtained using TRIzol RNA isolation system (Life Technologies, Grand Island, NY) according to the manufacturer's instruction.

Reverse transcription (RT) PCR was performed using HiScript III RT SuperMix for qPCR (R323-01; Vazyme, China). DNA was synthesized using 2 μg of RNA in 20 μl of reaction buffer containing 4 x

gDNA wiper mix and 5 × HiScript III qRT SuperMix.

Real-time PCR was performed on ABI PRISM 7000 Sequence Detection System (Applied Biosystems, Foster City, CA), using ChamQ SYBR qPCR Master Mix (High ROX Premixed) (Q341-02/03; Vazyme, China). The RNA levels of various genes were calculated after normalized by β-actin.

Transcriptomic analysis

RNA-seq was conducted to acquire the transcriptome of kidney tissues from various groups of mice. TRIzol reagent was utilized to extract total RNA, which was subsequently evaluated for RNA integrity using the RNA Nano 6000 Assay Kit of the Bioanalyzer 2100 system (Agilent Technologies, CA, USA). The total RNA served as input material for RNA sample preparations, with mRNA being purified from it using poly-T oligo-attached magnetic beads. Subsequently, cDNA was synthesized using the mRNA fragments as templates, and sequencing libraries were generated. The quality of the library was evaluated using the Agilent Bioanalyzer 2100 system, and the index-coded samples were clustered in accordance with the manufacturer's guidelines. Subsequently, the library preparations were sequenced on an Illumina Novaseq platform, generating 150 bp paired-end reads. The raw data, in fastq format, underwent initial processing via in-house perl scripts. The reference genome and gene model annotation files were obtained directly from the genome website. The reference genome's index was constructed through the utilization of Hisat2 v2.0.5, while the paired-end clean reads were aligned to the reference genome using the same software. The read numbers mapped to each gene were counted using FeatureCounts, and subsequently, the FPKM of each gene was computed based on the gene's length and the mapped read count. The DESeq2 R package (1.20.0) was employed to conduct differential expression analysis of two conditions/groups, each with two biological replicates. The clusterProfiler

R package was utilized to conduct Gene Ontology (GO) enrichment analysis on genes that were differentially expressed. Statistical enrichment of differential expression genes in KEGG pathways was tested using the same package. Reactome pathways were deemed significantly enriched by differential expressed genes if their corrected *P* value was less than 0.05. The local version of the GSEA analysis tool was employed to obtain GO, KEGG, Reactome, DO, and DisGeNET GSEA data sets.

Untargeted Metabolomics

Tissue samples were collected and prepared according to the manufacturer's instructions. UHPLC-MS/MS analyses were completed by an UHPLC (ThermoFisher, Germany) coupled with an Orbitrap Q ExactiveTM HF mass spectrometer (Thermo Fisher, Germany) in Novogene Co., Ltd. (Beijing, China). The raw data were processed by the Compound Discoverer 3.3 (CD3.3, ThermoFisher), including peak alignment, peak picking, and metabolite identification. The metabolite annotation was performed using the KEGG database, HMDB database and LIPIDMaps database. Pareto-scaled principal component analysis (PCA) and orthogonal partial least-squares discriminant analysis (OPLS-DA) were performed at meta X. Comparisons of metabolites were made by univariate analysis (t-test) between two groups. The metabolites with VIP > 1 and P-value < 0.05 and fold change ≥ 2 or FC ≤ 0.5 were considered statistically significant. Volcano plots and the correlation between differential metabolites were analyzed by R package (ropls).

Statistical analyses

All data were expressed as mean with SEM. Statistical analysis of the data was carried out using IBM SPSS Statistics 25. The validity of assumptions included the normality of data, homogeneity of variance

and independence of observations was assessed before performing statistical tests. Non-parametric tests were used when the assumptions of the statistical approach were not satisfied. Chi-square test was used for the comparisons of two rates or two composition ratios. For the parametric analysis, comparisons were made by Student's t-test for comparison of two groups, or via one-way analysis of variance followed by the Least Significant Difference or Dunnett's T3 procedure for comparison of multiple groups. A value of $P < 0.05$ was considered statistically significant. Bivariate correlation analysis was performed using Pearson and Spearman rank correlation analysis.

References

1. Chen Z, Tu Y, Zhang D, Liu C, Zhou Y, Li X, et al. A thermosensitive nanoplatform for photoacoustic imaging and NIR light triggered chemo-photothermal therapy. Biomater Sci. 2020; 8: 4299-4307.

2. Li J, Niu J, Min W, Ai J, Lin X, Miao J, et, al. B7-1 mediates podocyte injury and glomerulosclerosis through communication with Hsp90ab1-LRP5-β-catenin pathway. Cell Death Differ. 2022; 29: 2399-2416.

Supplementary Figures

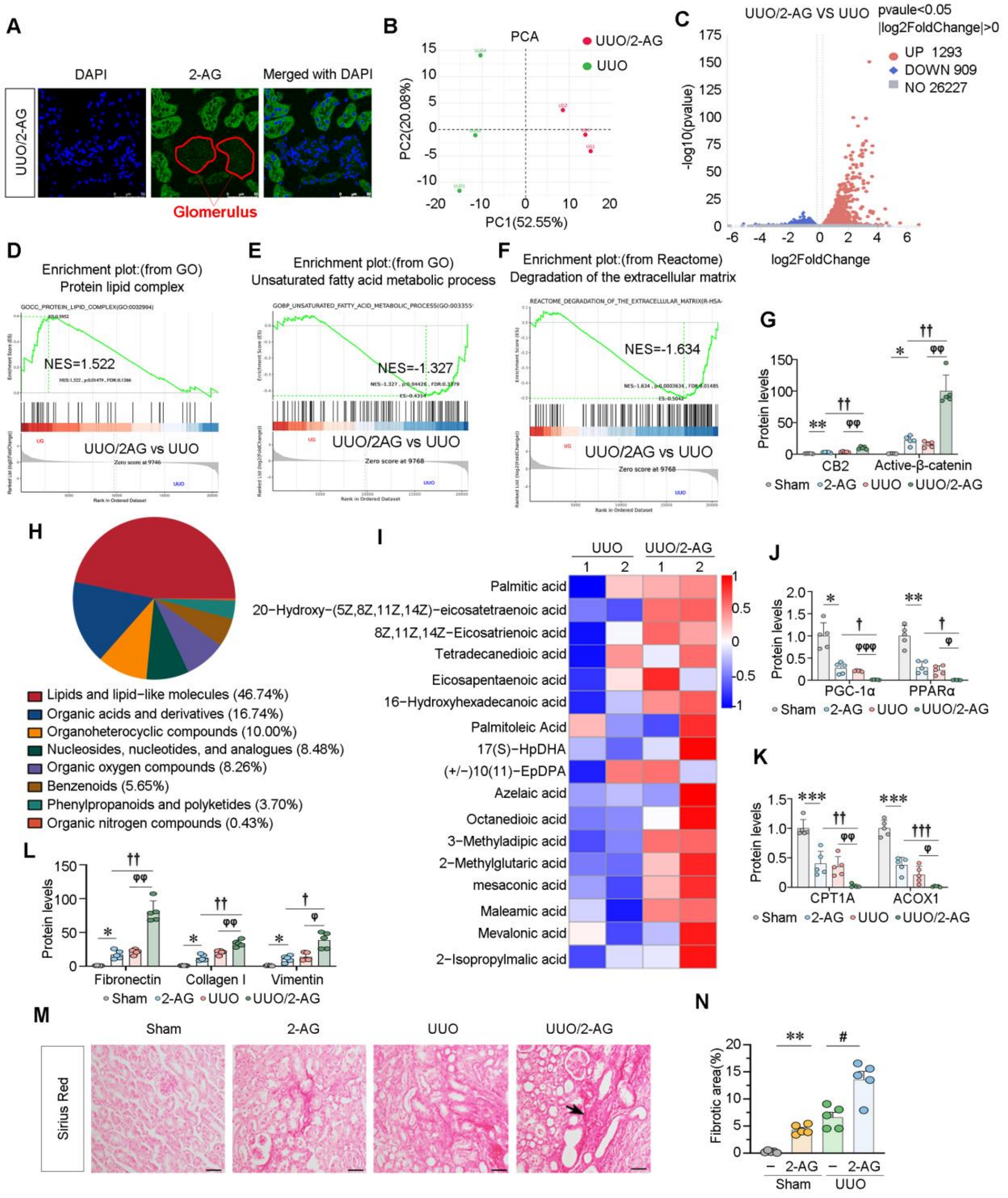

Supplementary Figure S1

Supplementary Figure S1. 2-AG exacerbates renal fibrosis and lipid accumulation in UUO mice

A. Representative micrographs showing the fluorescence of (indocyanine green)-2-AG in kidneys from UUO/2-AG mice. The area circled by the red line indicates glomerulus. Scale bar, 50 μm. **B.** Representative graph showing the principal component analysis (PCA) by RNA-seq in kidneys from UUO mice or UUO mice with intravenous injection of 2-AG. **C.** Volcano plot showing the differentially expressed genes in 2 groups. **D-F.** Gene set enrichment analysis (GSEA) enrichment plots showing

the different enrichment of genes in UUO/2-AG mice compared to UUO alone mice. $P < 0.05$. NES: normalized enrichment score. **G.** Quantitative data showing renal Expression of CB2 and Active-β-catenin in different groups. $^{*}P < 0.05$, $^{**}P < 0.01$, versus the sham control group alone; $^{\dagger\dagger}P < 0.01$ versus the 2-AG group alone; $^{\varphi\varphi}P < 0.01$ versus the UUO group alone. n = 5. **H**. Representative pie chart showing how many superclasses have been identified based on metabonomics. **I**. Representative heatmap plot of matabonomics analysis showing the changes in medium, long as well as very long-chain fatty acids and their derivatives. **J-L.** Quantitative data showing renal expression of PGC-1α, PPARα, CPT1A, ACOX1, Fibronectin, Collagen I and Vimentin in different groups. $^{*}P < 0.05$, $^{**}P < 0.01$, $^{***}P < 0.001$ versus the sham control group alone; $^{\dagger}P < 0.05$, $^{\dagger\dagger}P < 0.01$, $^{\dagger\dagger\dagger}P < 0.001$ versus the 2-AG group alone; $^{\varphi}P < 0.05$, $^{\varphi\varphi}P < 0.01$, $^{\varphi\varphi\varphi}P < 0.001$ versus the UUO group alone. n = 5. **M.** Representative micrographs showing the expression of fibrotic area in different groups. Arrow indicates positive staining. Scale bar, 50 μm. **N.** Quantitative data showing quantification of fibrotic area. Kidney sections were subjected to Sirius Red staining. At least 10 randomly selected fields were evaluated under 400 × magnification and results were averaged for each animal. $^{**}P < 0.01$ versus the sham control group alone; $^{\#}P < 0.05$ versus the UUO group alone. n = 5.

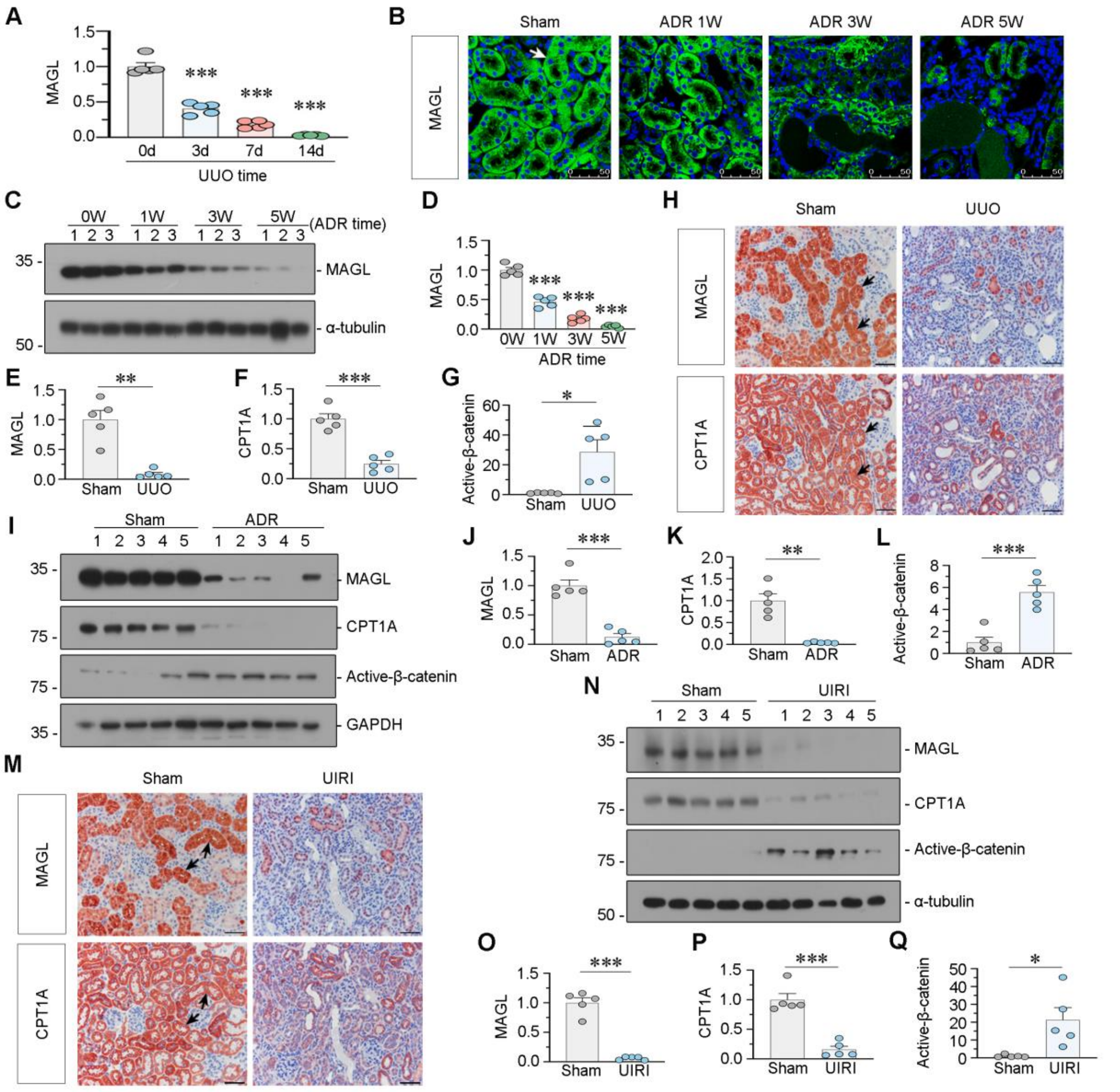

Supplementary Figure S2

Supplementary Figure S2. Loss of MAGL correlates with lipid accumulation and fibrosis

A. Quantitative data showing the expression of MAGL in different groups. Mice were sacrificed at different days after UUO surgery. Numbers (1 – 3) indicate each individual animal in a given group. ****P* < 0.001 versus the sham control group alone. n = 5. **B.** Representative immunofluorescence micrographs showing renal MAGL expression in different groups. Mice were sacrificed at different times after injection of ADR. White arrow indicates positive staining. Scale bar, 50 μm. **C-D.** Representative western blot and quantitative data showing the expression of MAGL in different groups. Numbers (1 – 3) indicate each individual animal in a given group. ****P* < 0.001 versus the sham group alone. n = 5. **E-G.** Quantitative data

showing the expression of MAGL, CPT1A and Active-β-catenin in UUO and sham mice. Numbers (1 – 5) indicate each individual animal in a given group. *P < 0.05, **P < 0.01, ***P < 0.001 versus the sham control group alone. n = 5. **H.** Representative micrographs showing renal MAGL (top) and CPT1A (bottom) expression in UUO and sham mice. Black arrows indicate positive staining. Scale bar, 50 μm. **I-L.** Representative western blot and quantitative data showing the expression of MAGL, CPT1A and Active-β-catenin in ADR and sham mice. Numbers (1 – 5) indicate each individual animal in a given group. **P < 0.01, ***P < 0.001 versus the sham control group alone. n = 5. **M.** Representative micrographs showing renal MAGL (top) and CPT1A (bottom) expression in UIRI and sham mice. UIRI mice were sacrificed at 11 d after ischemia reperfusion surgery. Black arrows indicate positive staining. Scale bar, 50 μm. **N-Q.** Representative western blot and quantitative data showing the expression of MAGL, CPT1A and Active-β-catenin in UIRI and sham mice. Numbers (1 – 5) indicate each individual animal in a given group. *P < 0.05, ***P < 0.001 versus the sham control group alone. n = 5.

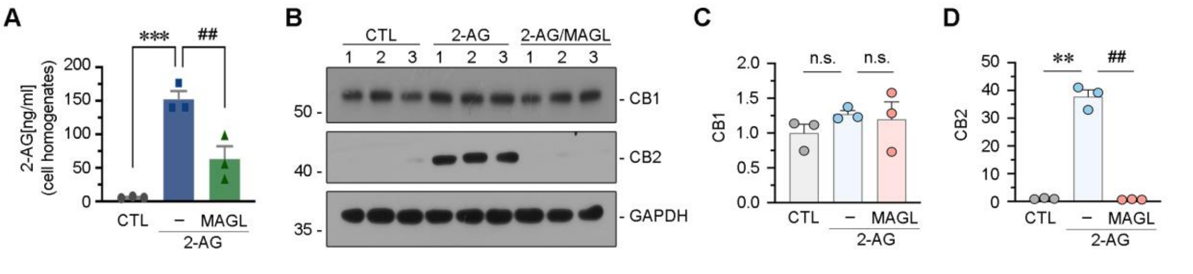

Supplementary Figure S3

Supplementary Figure S3. MAGL reduces 2-AG levels and inhibits CB2 expression in vitro

A. Representative graph showing 2-AG levels in cell homogenates in different groups by LC/MS analysis. HK-2 cells were pretreated with recombinant MAGL protein (100 ng/ml) for 1 h, and then treated with 2-AG (100 μM) for 24 h. ***P < 0.001 versus the control group alone. ##P < 0.01 versus 2-AG treatment group alone. **B-D.** Representative western blot and quantitative data showing the expression of CB1 and CB2 in different groups. Numbers (1 – 3) indicate each individual culture

in a given group. n.s., **$P < 0.01$ versus the control group alone. n.s., ##$P < 0.01$ versus 2-AG treatment group alone. n.s.: none of significance. n = 3.

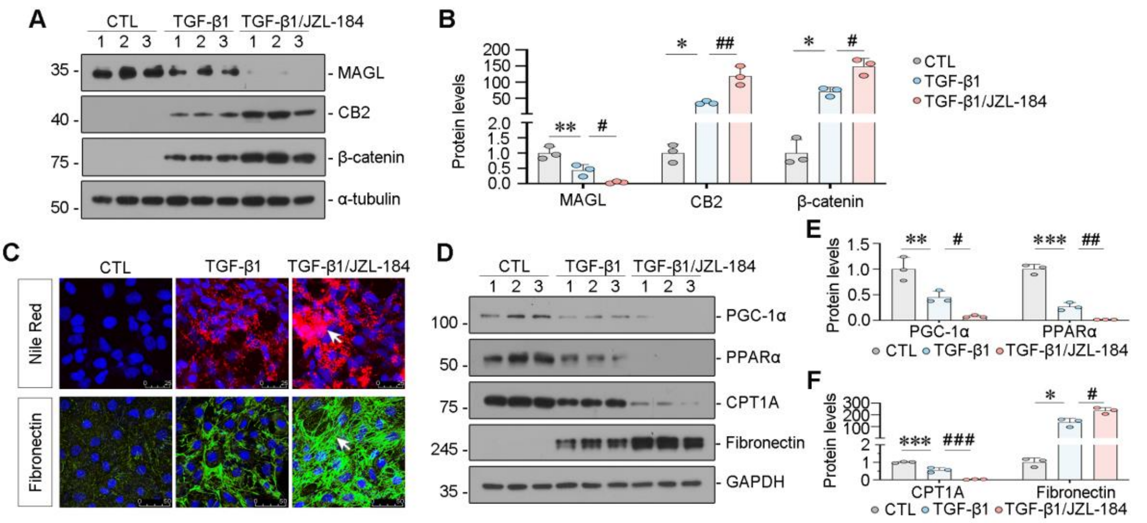

Supplementary Figure S4

Supplementary Figure S4. MAGL inhibitor exacerbates TGF-β1-induced lipotoxicity and fibrosis in renal tubular cells

A-B. Representative western blot and quantitative data showing the expression of MAGL, CB2 and β-catenin in different groups. HK-2 cells were treated with TGF-β1 (5 ng/ml) alone or cotreated with JZL-184 (20 μmol/ml) for 24 h. *$P < 0.05$, **$P < 0.01$ versus the control group alone; #$P < 0.05$, ##$P < 0.01$ versus the TGF-β1 treatment group alone. n = 3. **C.** Representative immunofluorescence micrographs showing the expression of lipid (Nile Red) and Fibronectin in different groups. White arrows indicate positive staining. For Nile Red staining, scale bar, 25 μm; For Fibronectin, scale bar, 50 μm. **D-F.** Representative western blot and quantitative data showing the expression of PGC-1α, PPARα, CPT1A and Fibronectin in different groups. *$P < 0.05$, **$P < 0.01$, ***$P < 0.001$ versus the control group alone; #$P < 0.05$, ##$P < 0.01$, ###$P < 0.001$ versus the TGF-β1 treatment group alone. n = 3.

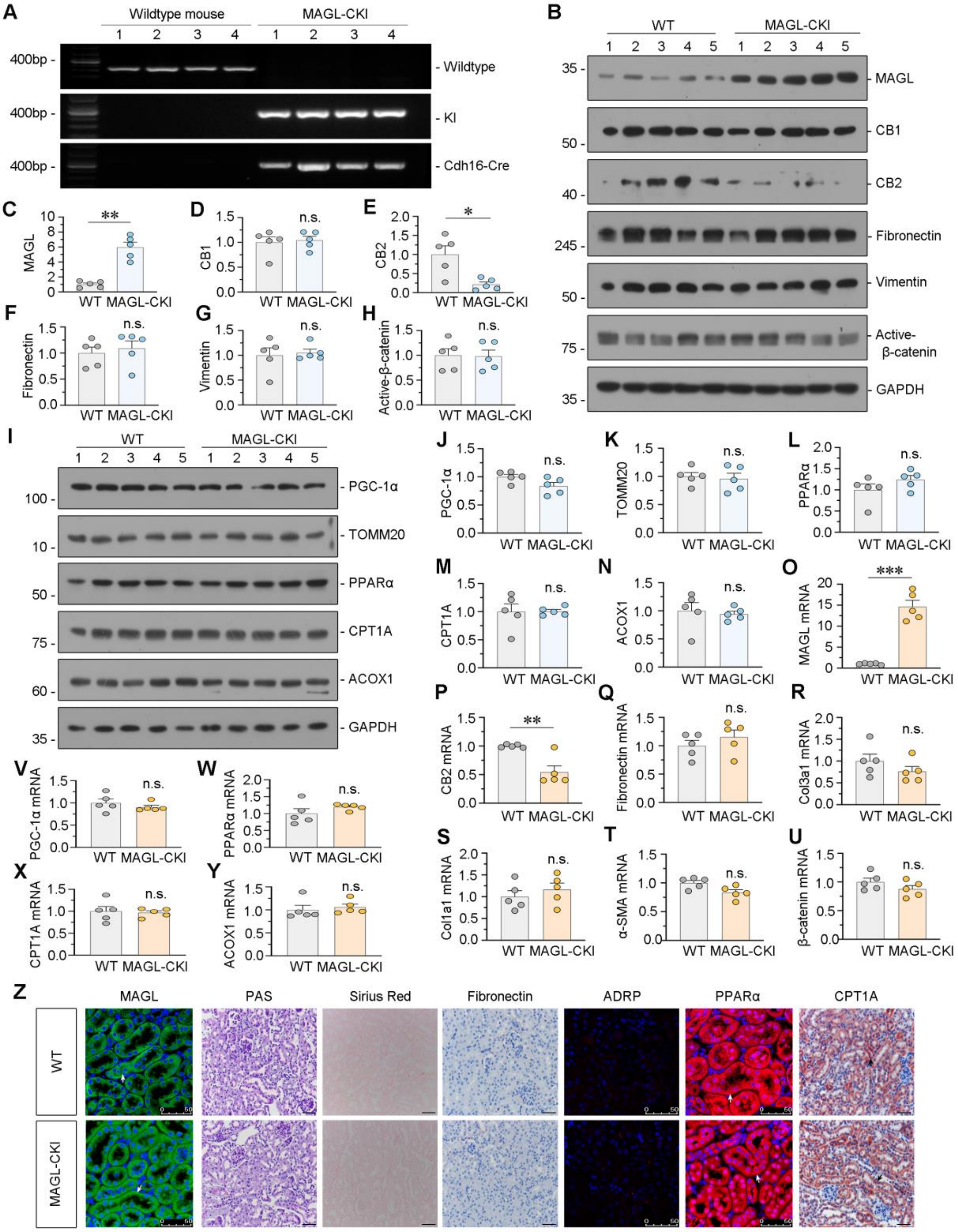

Supplementary Figure S5

Supplementary Figure S5. MAGL gene knock-in does not affect renal lipid accumulation, mitochondrial dysfunction, and fibrosis

A. Representative PCR analysis showing genotyping identification of tubular cell specific MAGL knock-in mice (Rosa26-

MAGL-CKI). **B-H.** Representative western blot and quantitative data showing the expression of MAGL, CB1, CB2, Fibronectin, Vimentin and Active-β-catenin in wildtype and MAGL-CKI mice. Numbers (1 – 5) indicate each individual animal in a given group. n.s., **P* < 0.05, ***P* < 0.01 versus the wildtype control group alone. n = 5. n.s.: none of significance. **I-N.** Representative western blot and quantitative data showing the expression of PGC-1α, TOMM20, PPARα, CPT1A and ACOX1 in wildtype and MAGL-CKI mice. Numbers (1 – 5) indicate each individual animal in a given group. n.s. versus the wildtype control group alone. n = 5. n.s.: none of significance. **O-Y.** Quantitative data showing the mRNA level of MAGL, CB2, Fibronectin, Col3a1, Col1a1, α-SMA, β-catenin, PGC-1α, PPARα, CPT1A and ACOX1 in wildtype and MAGL-CKI mice. n.s., ***P* < 0.01, ****P* < 0.001 versus the wildtype control group alone. n = 5. n.s.: none of significance. **Z.** Representative micrographs showing renal expression of MAGL, Fibronectin, ADRP, PPARα, CPT1A and PAS, Sirius Red staining in 2 groups. Arrows indicate positive staining. Scale bar, 50 μm.

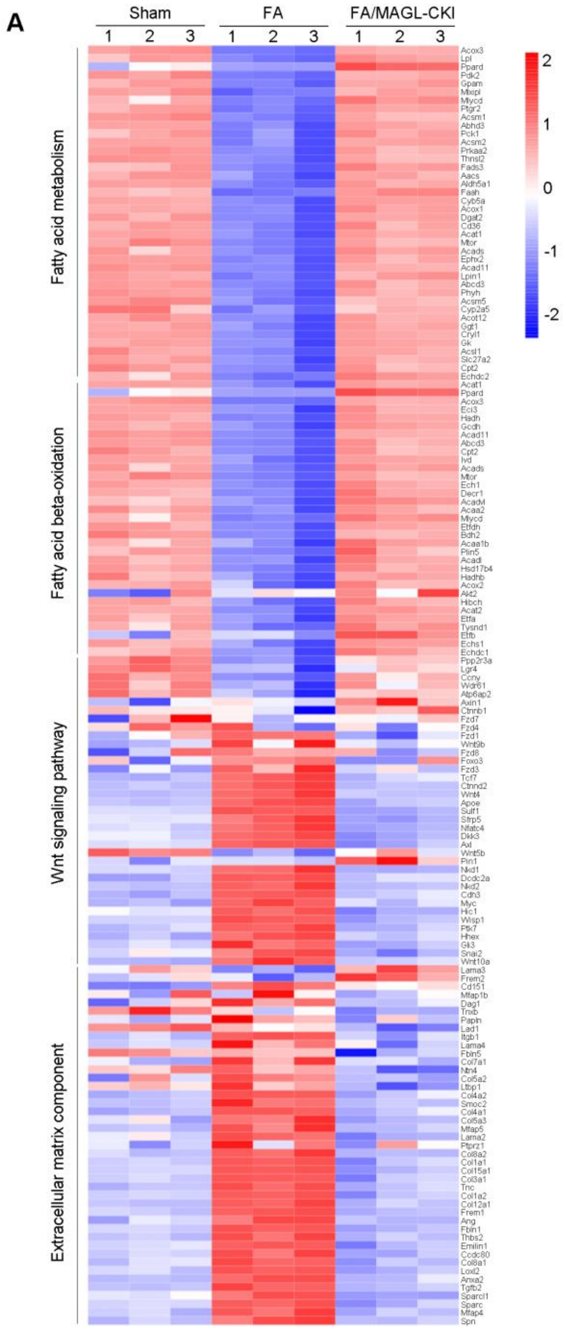

Supplementary Figure S6

Supplementary Figure S6. MAGL-CKI mice preserves the FAO function, mitochondrial biogenesis, and inhibit fibrosis

A. Representative heatmap plot of transcriptomic analysis showing the changes in Fatty acid metabolism, Fatty acid beta-oxidation, Wnt signaling pathway and Extracellular matrix in different groups.

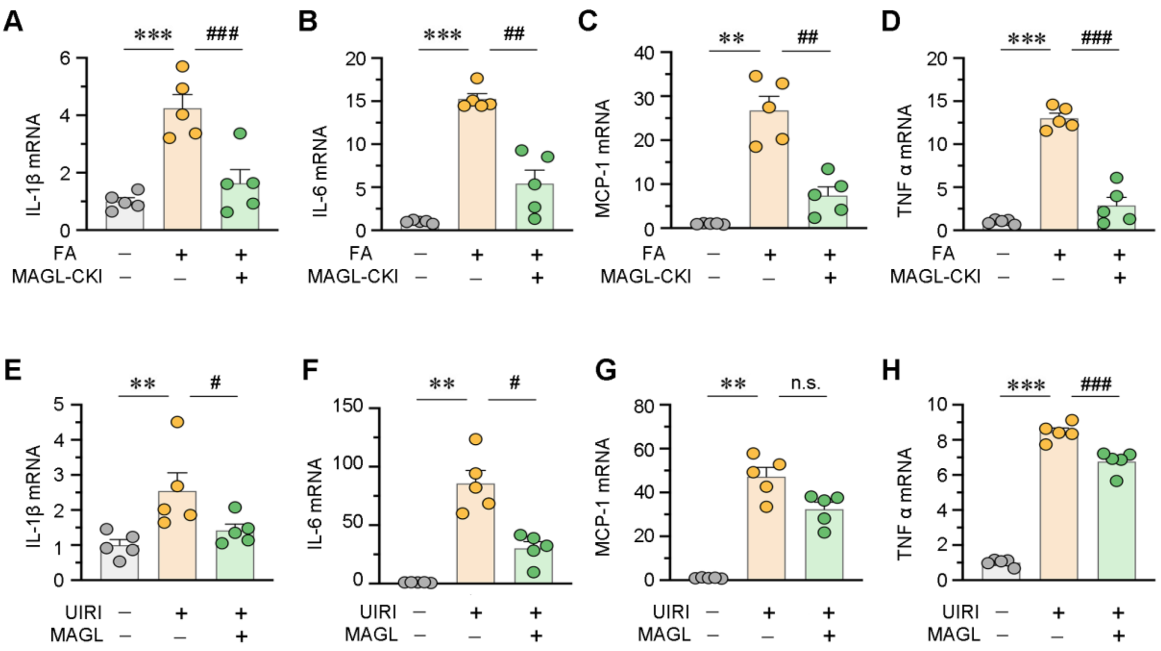

Supplementary Figure S7

Supplementary Figure S7. MAGL protects against inflammation

A-D. Quantitative data showing renal mRNA levels of IL-β, IL-6, MCP-1 and TNF-α in different groups. $^{**}P < 0.01$, $^{***}P < 0.001$ versus the wildtype control group alone; $^{\#\#}P < 0.01$, $^{\#\#\#}P < 0.001$ versus the FA treatment group alone. n = 5. **E-H.** Quantitative data showing renal mRNA levels of IL-β, IL-6, MCP-1 and TNF-α in different groups. $^{**}P < 0.01$, $^{***}P < 0.001$ versus the wildtype control group alone; n.s., $^{\#}P < 0.05$, $^{\#\#\#}P < 0.001$ versus the UIRI group alone. n = 5. n.s.: none of significance.

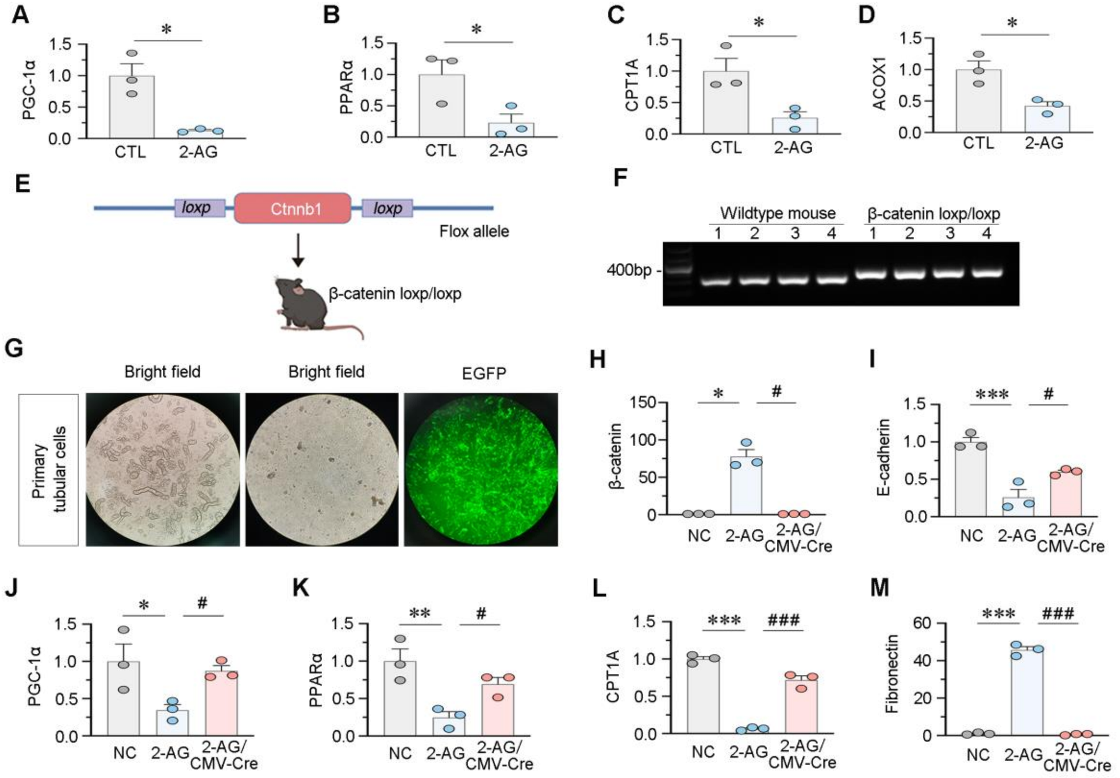

Supplementary Figure S8

Supplementary Figure S8. 2-AG suppresses PPARα/PGC-1α-mediated FAO via β-catenin signaling

A-D. Quantitative data showing the expression of PGC-1α, PPARα, CPT1A and ACOX1 in 2 groups. HK-2 cells were treated with 2-AG for 24 h. *P<0.05 versus the control group alone. n = 3. **E-F.** Representative graph showing establishment of β-catenin loxp/loxp mice. Genotyping was confirmed by PCR analysis. **G.** Representative micrographs showing the bright field and fluorescence field (EGFP) in primarily cultured tubular cells from β-catenin loxp/loxp mice (under 100 × magnification). The cells were transfected with Adv-CMV-Cre or Adv-NC virus and then treated with 2-AG (100 μM). **H-M.** Quantitative data showing the expression of β-catenin, E-cadherin, PGC-1α, PPARα, CPT1A and Fibronectin in different groups. *P < 0.05, **P < 0.01, ***P < 0.001 versus the Adv-NC group alone; $^{\#}P$ < 0.05, $^{\#\#\#}P$ < 0.001 versus the Adv-NC+2-AG group alone. n = 3.

Supplementary table S1.

Clinical sample source: Serum samples from healthy individuals and CKD patients

Healthy individuals

No.	Gender	Age
1	M	28
2	M	19
3	M	22
4	M	20
5	M	21
6	M	29
7	M	30
8	M	19
9	M	25
10	M	19
11	M	26
12	M	26
13	M	29
14	M	23
15	M	29
16	M	27
17	M	27
18	M	19
19	M	29
20	M	25
21	M	27
22	M	27
23	M	26
24	M	26
25	M	29
26	M	26
27	M	23
28	M	19
29	M	24
30	F	26
31	F	27
32	F	33
33	F	27
34	F	26

CKD patients at 5 stage

No.	Gender	Age	eGFR(ml/min/1.73m^2)
1	F	58	5.00
2	F	54	6.18

3	M	24	3.62
4	F	56	7.17
5	F	62	6.55
6	M	57	8.95
7	M	29	6.23
8	M	33	9.25
9	F	75	3.90
10	M	40	7.82
11	M	45	7.61
12	F	37	9.37
13	F	41	3.76
14	F	32	5.45
15	M	49	9.46
16	F	54	12.05
17	M	41	5.75
18	M	67	12.65
19	F	57	7.98
20	M	36	4.32
21	M	45	14.95
22	M	69	9.32
23	M	53	8.15
24	M	54	5.01
25	M	33	3.76
26	M	73	3.80
27	M	32	8.14
28	M	41	6.82
29	M	23	9.68
30	M	36	4.87
31	F	70	6.34
32	M	51	12.71
33	F	59	9.34
34	M	53	5.97
35	M	67	14.89
36	F	51	5.10
37	F	62	5.99
38	M	59	7.83
39	M	51	5.20
40	M	37	6.12
41	M	31	5.30
42	M	75	7.61
43	M	52	6.03
44	M	49	12.07

Data used in Figure 1A-C; F = Female; M = Male.

Supplementary table S2.

Clinical sample source: Renal biopsy tissue samples from CKD patients

CKD patients

No.	G stage	Gender	Age	eGFR(ml/min/1.73m^2)
1	CKD 1	F	28	129.08
2	CKD 1	F	51	91.13
3	CKD 1	F	27	121.99
4	CKD 1	F	47	108.57
5	CKD 1	F	48	108.49
6	CKD 1	M	25	126.95
7	CKD 1	F	22	126.35
8	CKD 1	F	27	140.63
9	CKD 1	M	40	112.88
10	CKD 1	M	41	108.31
11	CKD 1	M	24	108.43
12	CKD 1	F	44	108.26
13	CKD 1	F	24	130.95
14	CKD 1	F	39	92.43
15	CKD 1	F	57	103.17
16	CKD 1	F	29	120.29
17	CKD 1	M	24	123.41
18	CKD 1	M	35	101.79
19	CKD 1	F	48	110.63
20	CKD 1	F	25	107.44
21	CKD 1	M	50	107.83
22	CKD 1	F	49	97.68
23	CKD 1	M	22	105.49
24	CKD 1	F	28	111.1
25	CKD 1	F	42	106.61
26	CKD 1	F	43	115.37
27	CKD 1	M	45	102.1
28	CKD 1	F	36	113.23
29	CKD 1	F	23	90.9
30	CKD 1	F	34	116.14
31	CKD 1	F	29	121.71
32	CKD 1	F	40	110.71
33	CKD 1	M	48	94.25
34	CKD 1	M	36	112.18
35	CKD 2	F	53	66.26
36	CKD 2	F	40	83.21
37	CKD 2	F	50	81.38
38	CKD 2	M	40	63.49
39	CKD 2	M	55	88.45

40	CKD 2	M	34	72.65
41	CKD 2	M	64	76.46
42	CKD 2	M	28	81.87
43	CKD 2	F	57	88.97
44	CKD 2	M	50	72.63
45	CKD 2	M	63	89.98
46	CKD 2	F	70	89.17
47	CKD 2	F	35	76.47
48	CKD 2	F	33	86.05
49	CKD 2	F	33	77.55
50	CKD 2	M	53	75.52
51	CKD 2	F	58	76.93
52	CKD 2	F	41	80.1
53	CKD 2	F	28	83.89
54	CKD 2	M	45	63.17
55	CKD 2	M	33	81.84
56	CKD 2	F	51	68.34
57	CKD 2	M	63	71.28
58	CKD 3	F	50	48.26
59	CKD 3	M	46	36.68
60	CKD 3	F	25	34.38
61	CKD 3	F	44	55.6
62	CKD 3	M	38	50.98
63	CKD 3	F	18	39.22
64	CKD 3	M	44	32.2
65	CKD 3	F	48	48.43
66	CKD 3	M	68	40.63
67	CKD 3	M	38	52.7
68	CKD 3	M	45	57.3
69	CKD 3	M	37	43.11
70	CKD 3	F	48	41.37
71	CKD 3	F	22	41.17
72	CKD 3	F	35	48.44
73	CKD 3	F	41	56.14
74	CKD 3	F	47	51.47
75	CKD 3	M	55	39.09
76	CKD 4	M	56	26.45
77	CKD 4	M	48	21.89
78	CKD 4	M	49	23.88
79	CKD 4	F	67	27.19
80	CKD 4	M	32	17.45
81	CKD 4	F	34	23.74
82	CKD 4	M	45	25.75

83	CKD 5	M	51	13.33
84	CKD 5	M	54	2.61
85	CKD 5	M	38	6.21
86	CKD 5	M	59	4.66
87	CKD 5	F	50	5.32
88	CKD 5	M	56	8.32
89	CKD 5	M	83	11.66

Data used in Figure 3D & F; Figure 4D, G, H & I. F = Female; M = Male.

Supplementary table S3.

Clinical sample source: Urine samples from healthy individuals and CKD patients

Healthy individuals

No.	Gender	Age	Urinary MAGL (pg/mg)
1	F	26	341.0221632
2	F	33	894.642131
3	F	27	307.6385853
4	F	27	1081.704609
5	F	25	729.7321049
6	M	26	171.7834208
7	M	25	211.152718
8	M	25	311.3286571
9	M	28	142.6557255
10	F	24	129.3172601
11	F	27	504.9118286
12	F	30	154.9712778
13	F	24	150.0778726

CKD patients

No.	G stage	Gender	Age	eGFR(ml/min/1.73m^2)	Urinary MAGL (pg/mg)
1	CKD1	M	56	98.6	180.5193846
2	CKD1	F	47	105.4	6.104819106
3	CKD1	M	58	98.9	38.92618539
4	CKD1	M	48	97	39.54730818
5	CKD1	F	42	115.4	57.66846939
6	CKD1	M	63	96	20.82221815
7	CKD1	F	30	140.2	93.97456132
8	CKD1	F	47	105.4	91.10808055
9	CKD1	F	48	104.1	10.58855424
10	CKD1	F	52	97.5	38.95054793
11	CKD1	F	30	113.8	126.8174145
12	CKD1	F	21	129.5	38.05877534
13	CKD1	M	59	98.8	92.42660399

14	CKD1	M	46	109.5	81.71865385
15	CKD1	M	28	105.4	191.1578822
16	CKD1	M	22	123.1	154.480524
17	CKD1	M	43	100.5	60.46820352
18	CKD1	M	33	115.2	31.53836225
19	CKD1	M	48	94.2	68.35892165
20	CKD1	F	27	134.9	62.85161791
21	CKD1	F	64	96.4	114.9032194
22	CKD1	M	32	118.7	104.4532898
23	CKD1	M	46	105.2	76.96246288
24	CKD1	M	52	101.4	139.653226
25	CKD1	F	28	111.1	195.6812191
26	CKD1	M	45	109	137.3971158
27	CKD1	M	59	93.4	139.7311148
28	CKD1	F	34	110.6	42.53133655
29	CKD1	M	53	103	74.82220467
30	CKD1	M	44	106.6	44.35704113
31	CKD1	F	29	150.2	70.2072238
32	CKD1	F	17	135.7	104.8414661
33	CKD1	M	34	114.4	125.2002836
34	CKD1	F	27	134.9	149.5767598
35	CKD1	M	54	98.9	84.85976672
36	CKD1	F	49	106.4	87.05654941
37	CKD1	M	20	92.9	191.2244017
38	CKD1	M	26	135.7	147.2203605
39	CKD1	M	32	125.61	45.97638638
40	CKD2	M	48	89.1	118.7693769
41	CKD2	M	23	69.5	66.00569934
42	CKD2	F	51	68.3	127.6083514
43	CKD2	F	60	66	128.2346678
44	CKD2	M	28	80	35.80706494
45	CKD2	M	48	71.1	146.6034884
46	CKD2	F	52	60.9	80.62339517
47	CKD2	M	62	78.6	114.6808148
48	CKD2	M	34	79.4	24.08131631
49	CKD2	F	50	84.1	41.61903657
50	CKD2	F	28	60.8	138.3744988
51	CKD2	M	31	61.49	53.22295559
52	CKD2	M	41	63.05	61.55483988
53	CKD2	M	26	63.69	186.6606866
54	CKD2	M	57	68.34	8.036696247
55	CKD3	M	53	51.3	10.62484576
56	CKD3	F	44	40.3	16.00972937

57	CKD3	M	41	44.1	22.49448359
58	CKD3	F	61	34.6	58.43535525
59	CKD3	M	29	53.9	56.2131169
60	CKD3	F	53	36.9	34.35742308
61	CKD3	F	47	46.8	101.1871965
62	CKD3	F	58	48.2	122.3472384
63	CKD3	M	65	35.41	12.19461301
64	CKD3	F	58	36.86	193.8363425
65	CKD3	M	41	38.62	69.28638943
66	CKD3	M	52	39.36	48.51889749
67	CKD3	M	50	47.24	88.37050972
68	CKD3	M	54	47.9	13.42528568
69	CKD3	M	54	48.72	20.9967715
70	CKD3	M	76	48.99	38.65661369
71	CKD3	M	53	53.17	17.34653249
72	CKD3	M	54	54.3	99.81519059
73	CKD3	M	74	56.05	42.51611457
74	CKD3	M	66	58.65	112.9020511
75	CKD4	M	38	18.1	38.89501871
76	CKD4	F	31	28	77.26630048
77	CKD4	M	34	24.9	47.28028134
78	CKD4	M	48	15.96	30.97381583
79	CKD4	F	39	16.93	97.84359516
80	CKD4	F	39	20.08	46.11492229
81	CKD4	M	67	22.17	12.88928334
82	CKD4	F	55	23.2	46.68841113
83	CKD4	F	53	26.84	45.67943039
84	CKD4	M	78	27.92	48.02196528
85	CKD4	M	28	28.74	30.9668033
86	CKD4	M	72	28.75	39.64988151
87	CKD5	M	35	14.4	21.7809338
88	CKD5	F	58	3.36	98.28285494
89	CKD5	M	54	3.8	28.00405774
90	CKD5	M	70	4.72	45.88592914
91	CKD5	M	36	4.84	37.66568841
92	CKD5	M	29	5.11	86.72366017
93	CKD5	M	59	5.57	62.17962851
94	CKD5	M	63	5.57	36.494151
95	CKD5	F	36	6	13.62214403
96	CKD5	M	45	6.08	59.45721339
97	CKD5	M	40	7.12	12.75576065
98	CKD5	M	56	7.37	3.62017063
99	CKD5	F	51	7.6	101.2239338

100	CKD5	M	40	7.72	48.69562757
101	CKD5	M	37	7.79	37.32577402
102	CKD5	M	51	8.35	68.53457566
103	CKD5	M	25	8.81	6.067609929
104	CKD5	M	37	8.94	127.0484867
105	CKD5	M	57	9.02	71.47779154
106	CKD5	M	35	9.27	58.61299195
107	CKD5	M	30	9.85	22.19649102
108	CKD5	M	56	10.26	20.04661621

Data used in Figure 3E & G. F = Female; M = Male.

Supplementary table S4.

Nucleotide sequences of the primers used for RT-PCR or real-time PCR

**Gene**	**Primer Sequence 5' to 3'**	
	**Forward**	**Reverse**
CB2-mouse	TATGCTGGTTCCCTGCACTG	GAGCGAATCTCTCCACTCCG
MAGL-mouse	AGGCGAACTCCACAGAATGTT	ACAAAAGAGGTACTGTCCGTCT
PGC-1α-mouse	AGTCCCATACACAACCGCAG	CCCTTGGGGTCATTTGGTGA
PPARα-mouse	TGCAAACTTGGACTTGAACG	GATCAGCATCCCGTCTTTGT
CPT1A-mouse	GGTCTTCTCGGGTCGAAAGC	TCCTCCCACCAGTCACTCAC
ACOX1-mouse	CTTGGATGGTAGTCCGGAGA	TGGCTTCGAGTGAGGAAGTT
CPT2-mouse	CAATGAGGAAACCCTGAGGA	GATCCTTCATCGGGAAGTCA
ACOX2-mouse	TACCAACGCCTGTTTGAGTG	TTTCCAGCTTTGCATCAGTG
Fibronectin-mouse	ATGTGGACCCCTCCTGATAGT	GCCCAGTGATTTCAGCAAAGG
Col3a1-mouse	CTGTAACATGGAAACTGGGGAAA	CCATAGCTGAACTGAAAACCACC
Col1a1-mouse	GCTCCTCTTAGGGGCCACT	CCACGTCTCACCATTGGGG
α-SMA-mouse	GTCCCAGACATCAGGGAGTAA	TCGGATACTTCAGCGTCAGGA
β-catenin-mouse	ATGGAGCCGGACAGAAAAGC	CTTGCCACTCAGGGAAGGA
IL-1β-mouse	AACCTTTGACCTGGGCTGTC	AAGGTCCACGGGAAAGACAC
IL-6-mouse	AGGAGACTTCACAGAGGATACCA	TTCCACGATTTCCCAGAGAACAT

MCP-1	CCCACTCACCTGCTGCTAC	TTCTTGGGGTCAGCACAGA
TNF-α	TCGTAGCAAACCACCAAGTG	CCTTGAAGAGAACCTGGGAG
β-actin-mouse	CAGCTGAGAGGGAAATCGTG	CGTTGCCAATAGTGATGACC
